# Evaluation of the Gonadotoxicity of Cancer Therapies to Improve Counseling of Patients About Fertility and Fertility Preservation Measures: Protocol for a Retrospective Systematic Data Analysis and a Prospective Cohort Study

**DOI:** 10.2196/51145

**Published:** 2024-03-20

**Authors:** Michael von Wolff, Ariane Germeyer, Bettina Böttcher, Isotta Martha Magaton, Irene Marcu, Janna Pape, Nicole Sänger, Verena Nordhoff, Marie Roumet, Susanna Weidlinger

**Affiliations:** 1 Division of Gynaecological Endocrinology and Reproductive Medicine University Women’s Hospital Bern Switzerland; 2 Department of Gynaecological Endocrinology and Fertility Disorders University Women’s Hospital Heidelberg Germany; 3 Department of Gynaecological Endocrinology and Reproductive Medicine Medical University of Innsbruck Innsbruck Austria; 4 Department of Gynaecological Endocrinology and Reproductive Medicine University Women’s Hospital Bonn Germany; 5 Centre of Reproductive Medicine and Andrology Department of Clinical and Surgical Andrology University of Münster Münster Germany; 6 Clinical Trial Unit University of Bern Bern Switzerland

**Keywords:** fertility, fertility preservation, cancer, gonadotoxicity, FertiPROTEKT, FertiTOX, data analysis, cohort study, internet, platform, internet-based, data

## Abstract

**Background:**

Cytotoxic treatments such as chemo- and radiotherapy and immune therapies are required in cancer diseases. These therapies have the potential to cure patients but may also have an impact on gonadal function and, therefore, on fertility. Consequently, fertility preservation treatments such as freezing of gametes and gonadal tissue might be required. However, as detailed data about the necessity to perform fertility preservation treatment are very limited, this study was designed to fill this data gap.

**Objective:**

Primary objective of this study is to analyze the impact of cancer therapies and chemotherapies on the ovarian reserve and sperm quality. Secondary objectives are to analyze the (1) impact of cancer therapies and chemotherapies on other fertility parameters and (2) probability of undergoing fertility preservation treatments in relation to specific cancer diseases and treatment protocols and the probability to use the frozen gametes and gonadal tissue to achieve pregnancies.

**Methods:**

First, previously published studies on the gonadotoxicity of chemo- and radiotherapies among patients with cancer will be systematically analyzed. Second, a prospective cohort study set up by approximately 70 centers in Germany, Switzerland, and Austria will collect the following data: ovarian function by analyzing anti-Müllerian hormone (AMH) concentrations and testicular function by analyzing sperm parameters and total testosterone immediately before and around 1 year after gonadotoxic therapies (short-term fertility). A follow-up of these fertility parameters, including history of conceptions, will be performed 5 and 10 years after gonadotoxic therapies (long-term fertility). Additionally, the proportion of patients undergoing fertility-preserving procedures, their satisfaction with these procedures, and the amount of gametes and gonadal tissue and the children achieved by using the frozen material will be analyzed. Third, the data will be merged to create the internet-based data platform FertiTOX. The platform will be structured in accordance with the *ICD* (*International Classification of Diseases*) classification of cancer diseases and will be easily be accessible using a specific App.

**Results:**

Several funding bodies have funded this study. Ten systematic reviews are in progress and the first one has been accepted for publication. All Swiss and many German and Austrian ethics committees have provided their approval for the prospective cohort study. The study registry has been set up, and a study website has been created. In total, 50 infertility centers have already been prepared for data collection, which started on December 1, 2023.

**Conclusions:**

The study can be expected to bridge the data gap regarding the gonadotoxicity of cancer therapies to better counsel patients about their infertility risk and their need to undergo fertility preservation procedures. Initial data are expected to be uploaded on the FertiTOX platform in 2026.

**Trial Registration:**

ClinicalTrials.gov NCT05885048; https://clinicaltrials.gov/study/NCT05885048

**International Registered Report Identifier (IRRID):**

DERR1-10.2196/51145

## Introduction

### Background

**Figure 1:**
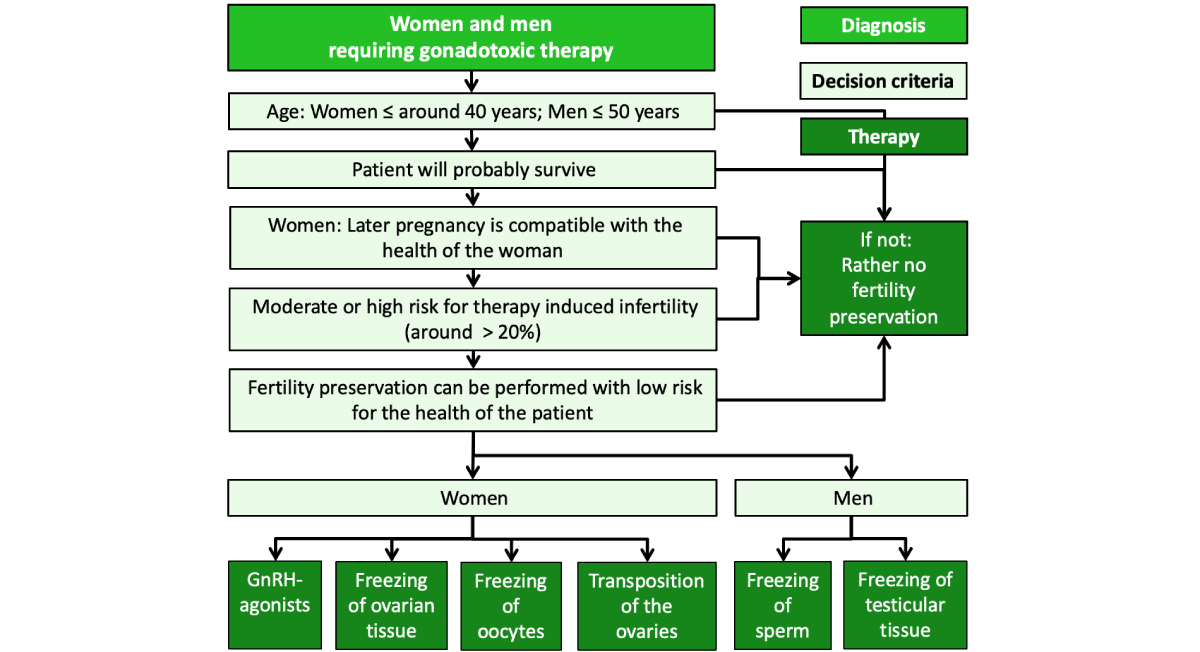
Algorithm for indicating fertility preserving therapies in women and men.

After reaching milestones in fertility preservation such as freezing of sperm and testicular tissue—and more recently, the first birth after transplantation of cryopreserved ovarian tissue [1], the introduction of luteal-phase random-start gonadotropin stimulation [2], and vitrification of oocytes [3]—fertility preservation treatments have been introduced in most countries, and fertility preservation has been accepted and defined as an important element to be considered before cancer treatments (Figure 1).

Medically, this has been evidenced by several national and international guidelines stating that fertility preservation counseling is required before administering gonadotoxic therapies [4-11], and this has been politically been shown as many countries have introduced reimbursement or coverage of fertility preservation treatments.

However, data about the gonadotoxicity of the numerous cancer treatment regimens are mostly very limited. Additionally, a recent study in mice has revealed that immune therapies such as checkpoint inhibitors have substantial impact on the ovarian reserve [12], but human data are not yet available.

Accordingly, indications for or against fertility-preserving therapies are not well defined with either the risk of overtreating patients or imposing unnecessary medical risks and burdens to the patients and, therefore, unnecessarily postponing the gonadotoxic therapies. However, the risk of undertreating patients with respective therapies imposes the risk of infertility, which can have a substantial impact on their quality of life after the onset of cancer [13].

Meanwhile, effective methods are available to reliably quantify the gonadotoxicity of cancer therapies. In women, ovarian function can be evaluated by analyzing anti-Müllerian hormone (AMH), follicle-stimulating hormone (FSH), luteinizing hormone (LH), and estradiol (E2) concentrations, and in men, testicular function is evaluated by analyzing sperm counts and total testosterone, FSH, and LH concentrations.

Even though these functional parameters exist, prospective and systematic short- and long-term data regarding the impact of specific cancer therapies on fertility based on these parameters are limited. It can be assumed that this is due to a lack of effective fertility preservation network structures in most countries. However, in Switzerland, Germany, and Austria, network structures that permit a systematic and continuous large-scale data analysis have been established.

Data should be made available as an easily accessible internet-based platform that is merged with already published data on the gonadotoxicity of cancer therapies and other relevant data such as the 5-year survival rates of the cancer diseases. The data platform will support physicians and other experts as well as patients in counseling about fertility risks imposed by cancer treatments and the necessity to undergo fertility-preserving measures.

We, therefore, designed a study to collect data on the gonadotoxicity of cancer therapies by systematically analyzing already published data and by setting up an international multicenter prospective cohort study to collect data on the gonadotoxicity of chemotherapies, radiotherapies, and immune therapies among men and women and to upload summarized data on an internet-based data platform.

### Objectives

The primary objective is to determine whether cancer therapies and specific chemotherapy protocols reduce AMH concentrations in women and sperm quality in men.

Secondary objectives are to analyze the impact of cancer therapies and chemotherapies on other fertility parameters such as the probability of undergoing fertility-preserving treatments in relation to the type of cancer disease and specific treatment protocols, the number of children, the future wish to have children, and age. Further objectives are to assess satisfaction with the decision to have undergone fertility-preserving procedures, the proportion of women and men who use their frozen gametes to achieve a pregnancy, the effect of different gonadotoxic therapies on long-term fertility, and the quality of life after the administration of cancer therapies. 

## Methods

### Study Design

The study consists of 2 parts: first, a series of systematic reviews; second, an international multicenter prospective exploratory observational cohort study of fertility-related parameters with a long-term follow-up of the fertility of patients with cancer in university and nonuniversity public hospitals and private infertility centers in Germany, Switzerland, and Austria. All data will be uploaded on an internet-based data platform.

### First Part: Retrospective Systematic Data Analysis

A series of systematic reviews will be performed in Switzerland, one of which will be performed in Germany. The reviews encompass diseases for which a sufficient number of studies has been published to perform a systematic review and which are relevant regarding fertility and fertility-preserving issues. The following reviews were initiated in 2023 and 2024 or are expected to be published in 2024 and 2025. They are registered in PROSPERO:

- A systematic review of the gonadotoxicity of Osteoscarcoma and Ewing´s sarcoma chemotherapies in postpubertal females and males: CRD42023331654, accepted for publication (*Journal of Adolescent and Young Adult Oncology*).
- Effects of chemotherapy and radiation in male genital organ tumors on ovarian and testicular function and fertility: CRD42023384057, submitted for publication in 2024.
- Effects of chemotherapy and radiation in mesothelial and soft tissue cancer on ovarian and testicular function and fertility: CRD42023385402, to be submitted for publication in 2024.
- Effects of chemotherapy, radiation and immunotherapy in breast cancer on ovarian and testicular function and fertility: CRD42023384042, to be submitted for publication in 2024.
- Effects of bone marrow transplantation in hematological cancers on ovarian and testicular function and fertility: CRD42023486928, to be submitted for publication in 2024.
- Effects of chemotherapy and radiation in eye, brain and central nervous system cancer on ovarian and testicular function and fertility: CRD42023385408, to be submitted for publication in 2024.
- Effects of chemotherapy and radiation in Hodgkin lymphoma on ovarian and testicular function and fertility: CRD42023384052, to be submitted for publication in 2025.
- Effects of chemotherapy and radiation in Non-Hodgkin lymphoma on ovarian and testicular function and fertility: CRD42024511940, to be submitted for publication in 2025.
- Effects of chemotherapy and radiation in colorectal cancer on ovarian and testicular function and fertility: CRD42024511944, to be submitted for publication in 2025.
- Effects of chemotherapy and radiation in leukemia on ovarian and testicular function and fertility: CRD42024511946, to be submitted for publication in 2025.

### Second Part: Prospective Cohort Study

The cohort study will collect data on the ovarian reserve and sperm from patients undergoing gonadotoxic therapies. Data collection was initiated on December 1, 2023, and will be continued at least for 5 years to analyze short-term fertility. Long-term fertility data will be collected until at least 2036. An initial version of the FertiTOX internet-based platform is expected to be set up in 2026. The trial is registered in ClinicalTrials.gov (NCT05885048).

### Inclusion Criteria

Each center that counsels patients with cancer about fertility issues and can also provide fertility-associated parameters may participate. A prerequisite requirement is that each center has received ethical approval for the study. Even though the study is intended to mainly include FertiPROTEKT network centers [14] and FERTISAVE [15] networks in Germany, Switzerland, and Austria, any other center worldwide can participate if the inclusion criteria are fulfilled.

Women and men aged 14-50 years (18-50 years in Germany due to national regulations) undergoing cancer therapies using chemotherapy, radiotherapy, or immune therapy (Figure 1) will be included.

### Recruitment and Informed Consent Procedure

Patients are recruited by reproductive physicians who are associated with the participating infertility centers. Approximately 70 centers (44 in Germany, 21 in Switzerland, and 6 in Austria) will participate in the study and will collect data (see Multimedia Appendix 1).

Patients who need gonadotoxic therapies will be counseled before the onset of the respective therapies. During counseling, patients are screened for their eligibility to be included in the study.

Furthermore, patients will be provided counseling forms to provide informed consent before the onset of gonadotoxic therapy to collect specific basic data and data on gonadal function from patients with cancer. Data will also be collected 12-15 months and 5 and 10 years after the end of gonadotoxic therapy.

The study participants will be informed that they will be contacted by the fertility center or a defined coworker of the study by telephone, email, or post to collect the respective data after the administration of gonadotoxic therapy.

### Study Registry

Data will be collected using the REDCap (Research Electronic Data Capture) software, a secure web application for building databases [16]. The REDCap registry has been set up and optimized by the study consortium with the support of the Clinical Trials Unit in Bern, REDCap technicians, and statisticians. The contents of the study registry (consultation before and 12-15 months after the end of the gonadotoxic therapy) are shown in the paper version of the registry (Multimedia Appendices 2-5). The participating centers will add the data to the registry without adding any definite identifiers such as name and date of birth. The participating centers can only see their patients. Patients can be traced with an individual code that is safeguarded by the centers. Only very few authorized persons will be provided access to all data in order to assess the data quality and to remind the centers to invite the patients for follow-up consultations.

### End Points

The primary end points are the AMH concentration in women and sperm count before and after gonadotoxic treatments. End points will be assessed before and 12-15 months after the end of the gonadotoxic treatments (patients with melanoma receiving adjuvant checkpoint inhibitor treatment will be evaluated every 3 months).

Secondary end points are FSH, LH, and E2 concentrations in women and total testosterone, FSH, and LH concentrations, total sperm count, and sperm motility in men. Further secondary outcome parameters are the number of patients who freeze gametes and gonadal tissue and satisfaction with the decision to have undergone fertility-preserving measures.

Long-term end points to be determined 5 and 10 years after the end of the gonadotoxic therapy are the abovementioned hormone and sperm parameters as well as the number of patients who become pregnant after gonadotoxic therapies spontaneously or after the use of frozen gametes and gonadal tissue.

### Sample Size Calculation

A power calculation was performed to determine whether the expected number of patients is sufficient to detect an effect of cancer treatment on fertility with a reasonable power.

The calculation was performed for the primary outcome (ie, sperm count for men and AMH concentration for women) for men and women separately. Calculations were performed within each cancer entity and for each specific treatment protocol separately with a paired *t* test using Stata (release 17.0; StataCorp).

We set the effect size so that a relative risk of 50% was not missed. For the sperm count, where the mean value in healthy men is 64 million (SD 47 million) [17], this would correspond to an effect size of 0.67. For AMH concentration in women, where means and SDs can be assumed to be equal [18], it would correspond to an effect size of 0.5. The intraindividual correlation was set to 0.5 [17,18]. To account for multiple testing (analysis will be performed with 43 treatment groups for men and women separately), the Šidák correction was used, and the significance level was adjusted to 0.0006 (2-sided).

Table 1 shows that a sufficient power of >80% will be reached for most treatments. If the number of cases was twice as high and the intrapatient correlation coefficient was 0.7, a power of >80% would be reached for all treatment levels. As the study is expected to be extended, a sufficient number of patients can be expected in order to reach this goal.

**Table 1:** Expected number of cases and resulting power reached per specific treatment (treatment protocols) in the most common *ICD (International Classification of Diseases)* cancer groups [19].

*ICD* cancer group	Cases, n^a^	Specific treatments (treatment protocols), n	Effect size (females/ males)^b^	Power (females/ males)^b^, %/%
Breast	2000	6	0.5/—^c^	100/—
Hodgkin lymphoma	1000	2	0.5/0.68	100/100
Bone and articular cartilage	400	3	0.5/0.68	98.6/100
Female genital organs	350	3	0.5/—	96.5/—
Male genital organs	2000	4	—/0.68	—/100
Digestive organs	200	7	0.5/0.68	14.3/41.4
Mesothelial and soft tissue	200	3	0.5/0.68	67.2/96.7
Eye, brain, and central nervous system	300	2	0.5/0.68	99.5/100
Non-Hodgkin lymphoma	300	8	0.5/0.68	26.2/64.4
Leukemia	200	5	0.5/0.68	30.6/70.8

^a^The estimation of the number of cases is based on the number of patients previously counseled and documented in the FertiPROTEKT and FERTISAVE registries.

^b^Power is calculated for each treatment protocol for men and women separately. The number of patients per protocol is calculated as the number of cases divided by the number of treatment protocols. The sex ratio is assumed to be 1:1, except for cancer of the breast and genital organs.

^c^Not available.

### Data Analysis

The impact of cancer therapies and chemotherapies on the primary outcomes will be assessed for each *ICD* cancer group [19] and for each specific treatment regimen separately by comparing values measured before and after the gonadotoxic therapy using paired *t* tests. *P* values will be adjusted for multiple testing using the false discovery rate controlling procedure.

Consequences on other fertility parameters measured before and 12-15 months after the end of gonadotoxic therapy (ie, AMH, FSH, LH, and E2 concentrations in women and total testosterone, FSH, and LH concentrations and total sperm count, sperm concentration and motility in men) will be assessed using the same methodology, but without false discovery rate adjustment. Long-term consequences will be investigated later by comparing values measured before and 5 and 10 years after the gonadotoxic therapy. However, due to the long period until the final analysis, statistics might be adapted in relation to future development of cancer and fertility-preserving therapies.

As age was identified to potentially modify the effect of gonadotoxic therapy on female fertility, we will additionally conduct stratified analyses and document the impact of gonadotoxic therapy in the different female age groups.

Finally, the effects of different cancers, treatment protocols, age, and the presence of the patients’ children before cancer therapy on primary and secondary fertility outcomes will be analyzed using linear mixed effect models.

The frequency of patients undergoing fertility-preserving treatments, namely freezing of ovarian tissue, oocytes and embryos, testicular tissue, and sperm will be calculated for each *ICD* cancer group and for each specific treatment protocol separately and additionally presented with the associated 95% Wilson CI. Potential effects of the patients’ characteristics on the abovementioned binary outcomes will be analyzed using logistic regression analysis.

### Consortium of Experts Involved in the Study

**Figure 2:**
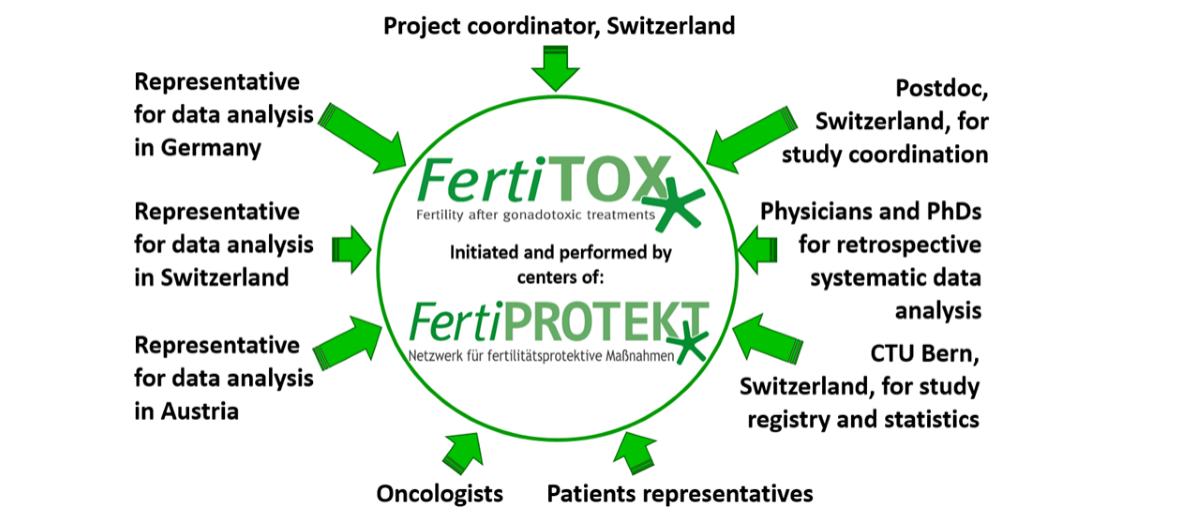
The consortium of experts who control the project.

The study is supervised by several experts in Germany, Switzerland, and Austria (Figure 2). Each country provides the logistics, manpower, and experts to collect and control data collection and analysis. Interpretation of data will be supported by oncologists. Data are added to a REDCap study registry, which is provided by the Clinical Trial Unit in Bern, Switzerland, which is also responsible for statistical analysis. The data platform will be programmed by an IT company, and data presentation will be optimized by the involved experts and by the patients’ representatives. The data platform, called “FertiTOX,” will be part of the network “FertiPROTEKT.”

### The FertiTOX Platform

**Figure 3:**
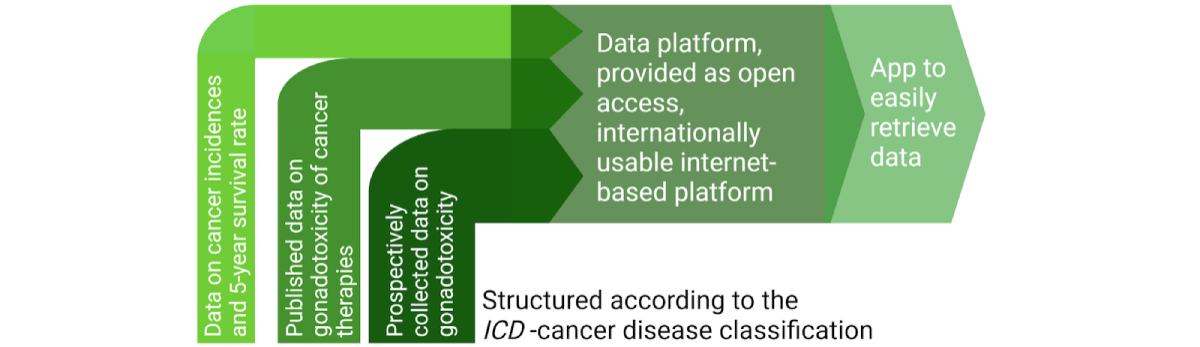
The internet-based platform FertiTOX that will be used to distribute the data. *ICD: International Classification of Diseases*.

The FertiTOX platform is an internet-based information platform that will be accessible to everybody and will be structured on the basis of the *ICD* cancer disease groups (Figure 3). The brand name FertiTOX has been registered and the domains will be used. A study website [20] has been set up.

Data of women and men will be presented on the FertiTOX platform in 3 hierarchies:

- First hierarchy: presentation of data regarding incidences and 5-year survival according to the *ICD-*classified cancer disease.
- Second hierarchy: presentation of already published data regarding gonadotoxicity and fertility preservation issues regarding specific *ICD-*classified diseases.
- Third hierarchy: presentation of prospective short-term and long-term data on the ovarian reserve and sperm quality before and after gonadotoxic therapies in accordance with *ICD-*classified diseases.

Once the internet-based platform is successfully established, a platform-specific app will be developed to allow easy retrieval of data.

### Dissemination

Data about the progress of the study will be disseminated through a study website [20]. The findings will be propagated through journal articles, conference presentations, and the FertiTOX platform.

### Ethical Considerations

Ethical approval has been granted by the relevant ethics committees. In Switzerland, all 7 cantonal ethical committees have provided consent for all 21 participating centers. In Germany, the ethics committee in Heidelberg and in Austria, the committee in Innsbruck were chosen as the national leading ethical committees. Based on the ethical votes approved by these 2 committees, all other participating centers in Germany and Austria submitted an ethics application to their ethical committee.

Written consent was obtained from the participants. The participants have been informed about the processing of data and their rights. The participants also provided written consent for anonymized data to be transferred to other registries for further analysis if the other registries have the same high security standards as the REDCap registry. The data are collected in a REDCap study registry that complies with several data security and confidentiality conditions. Specific identifiers are not added to the registry; only the year of birth is added. The data are stored in Bern, Switzerland.

The participating centers can only access the patient’s data that they have added to the registry. The patients can be identified with an individual code generated by the REDCap system, which is locked in a safe place by the investigator of the participating center. Traceability of the data is ensured by these identification codes. Patients provide permission to be followed up. Patients are only contacted by the center or by authorized personnel employed by the principal investigator or the national representatives of the study. Statistical analysis will be based on anonymized data.

No compensation will be offered to the patients (potential users of the internet-based platform) for participating in the study.

## Results

As of February 18, 2024, the following has been accomplished.

The first systematic review analyzing the impact of cancer therapies on bone tumors has been accepted for publication (in the *Journal of Adolescent and Young Adult Oncology*). Nine more systematic reviews have been initiated, encompassing the impact of cancer therapies on fertility in testicular cancer; soft tissue cancer; breast cancer; eye, brain, or central nervous system cancer; Hodgkin lymphoma; non-Hodgkin lymphoma; rectal or sigmoid cancer; leukemia; and on bone marrow transplantation.

The prospective cohort study has been prepared. All Swiss ethics committees and the leading ethics committees in Germany and in Austria have provided their approval. In total, 70 infertility centers (Multimedia Appendix 1) have started to be prepared for data collection. The study registry has been set up and activated, and a study website [20] has been created.

Funding for the study has been obtained from several funding bodies, which were not involved in the study’s conceptualization, analysis, and data dissemination.

Recruitment of the patients started on December 1, 2023, and initial data are expected to be uploaded on the FertiTOX platform in 2026.

## Discussion

### Anticipated Findings

The first systematic reviews we have already carried out have revealed that data on the impact of gonadotoxic therapies on fertility are quite comprehensive in some diseases such as breast and testicular cancers and Hodgkin lymphoma. For these diseases, a meta-analysis will also be carried out to analyze the overall effect of oncological therapies on fertility. Furthermore, for these diseases, the gonadotoxic effect of specific chemotherapy protocols on fertility will also be assessed. For the other diseases, data are very poor, and outcome parameters will probably need to be limited to a rather vague outcome parameter defined as “suspected infertility.” Long-term fertility data based on the ovarian reserve and sperm parameters and on spontaneous conceptions and conceptions using cryopreserved gametes or gonadal tissues are very poor or nonexistent in all kinds of cancers.

This finding supports the necessity to perform the large prospective cohort studies, as described in this paper. This cohort study has already successfully been set up and data collection was initiated on December 1, 2023.

Robust data on the gonadotoxicity of different treatment regimens, mainly in cancer therapies, is the last major deficit in fertility preservation. All other requirements have been established in many countries, such as fertility preservation procedures and specialized centers to counsel patients and to perform these procedures. Furthermore, many oncologists have been sensitized to address the fertility risk of cancer therapies and the possibility to perform fertility preservation procedures.

However, comprehensive data about the specific gonadotoxicity of different treatment regimens required to counsel patients and to decide if fertility preservation measures should be recommended or not are still missing.

Previous studies have addressed fertility issues in patients with cancer and the impact of cancer therapies on fertility, but these studies are mostly based on spontaneous pregnancies following cancer therapies or are based on data such as the onset of puberty, cycle regularity, and low sperm counts. Studies based on pre- and postchemotherapy levels of AMH and sperm counts are very limited and are mainly limited to female patients with breast cancer [21-24].

To bridge this data gap, we designed a study to analyze published data and data that will be collected in at least 3 countries by centers belonging to the FertiPROTEKT network. The data will be analyzed und disseminated via the FertiFOX web-based platform to provide patients, researchers, and clinicians access to the analysis, which will be graphically illustrated. By including patients’ representatives in the data analysis, we will ensure that the data are presented in a manner that is also understandable to patients. The data will not only be presented as a single curve presenting the means and showing odds ratios and CIs, which presents the average impact of therapies on gonadal function, but also show individual variations by presenting a set of curves.

The study has been designed to follow up on patients for at least 10 years. Such a long follow-up will enable us to evaluate how many patients have undergone fertility-preserving procedures but unfortunately did not survive. Furthermore, we will be able to estimate how many patients conceived spontaneously or by using the frozen gametes and gonadal tissue. Such an analysis is essential to evaluate the long-term efficacy of fertility preservation counseling and treatment. We are aware that some patients might be lost to follow-up. However, as the number of included patients is expected to be very high, it can be assumed that the amount of data will still be sufficiently high.

### Limitations

A limitation of this study is that data are mainly collected in Germany, Switzerland, and Austria. Accordingly, data will not be available for other treatment regimens performed elsewhere. However, as other countries are also invited to participate in this study, this limitation could possibly be reduced. Another limitation is that children will not be evaluated primarily because the study is based on AMH values and sperm parameters, which can hardly be interpreted in (AMH concentration) or cannot be collected from (sperm parameters) children.

The last limiting factor is that new therapies are evolving very fast and that new combination therapies combining conventional chemotherapy agents and immune therapies will be developed, which will increase the complexity of our data analysis. However, it is expected that this limitation can at least in part be compensated by the prospective design of the study, which will include a higher number of new therapies. Furthermore, the impact of checkpoint inhibitors, which have been shown to be gonadotoxic in female mice, will be analyzed separately in women with melanoma undergoing adjuvant therapy with checkpoint inhibitors. These results can be used to better estimate the gonadotoxicity of combination therapies involving checkpoint inhibitors.

### Conclusions

In conclusion, the study can be expected to bridge the data gap regarding the gonadotoxicity of several therapies and gonadotoxic treatment regimens to better counsel patients regarding their infertility risk and their need to undergo fertility-preserving procedures.

## Appendix

Multimedia Appendix 1 Centers participating the prospective cohort study (in alphabetical order).

Multimedia Appendix 2 REDCap registry: Documentation in females before gonadotoxic therapy.

Multimedia Appendix 3 REDCap registry: Documentation in males before gonadotoxic therapy.

Multimedia Appendix 4 REDCap registry: Documentation in females 12-15 months after the end of gonadotoxic therapy.

Multimedia Appendix 5 REDCap registry: Documentation in males 12-15 months after the end of gonadotoxic therapy.

## Abbreviations

AMH: anti-Müllerian hormone
E2: estradiol
FSH: follicle-stimulating hormone
*ICD*: *International Classification of Diseases*
LH: luteinizing hormone
REDCap: Research Electronic Data Capture

## Data Availability

Participants have provided written consent that the data can also be analyzed by others if sufficient data safety can be confirmed. Accordingly, data can be considered by the study consortium to be transferred to others if sufficient data safety has been proven.

## References

[1] Donnez J, Dolmans M M, Demylle D, Jadoul P, Pirard C, Squifflet J, Martinez-Madrid B, van Langendonckt A (2004). Livebirth after orthotopic transplantation of cryopreserved ovarian tissue. Lancet.

[2] Cobo Ana, Meseguer Marcos, Remohí José, Pellicer Antonio (2010). Use of cryo-banked oocytes in an ovum donation programme: a prospective, randomized, controlled, clinical trial. Hum Reprod.

[3] von Wolff M, Thaler CJ, Frambach T, Zeeb C, Lawrenz B, Popovici RM, Strowitzki T (2009). Ovarian stimulation to cryopreserve fertilized oocytes in cancer patients can be started in the luteal phase. Fertil Steril.

[4] Oktay Kutluk, Harvey Brittany E, Partridge Ann H, Quinn Gwendolyn P, Reinecke Joyce, Taylor Hugh S, Wallace W Hamish, Wang Erica T, Loren Alison W (2018). Fertility preservation in patients with cancer: ASCO Clinical Practice Guideline Update. J Clin Oncol.

[5] (2022). Fertilitätsprotektion bei onkologischen Erkrankungen.

[6] Practice Committee of the American Society for Reproductive Medicine. Electronic address: asrm@asrm.org (2019). Fertility preservation in patients undergoing gonadotoxic therapy or gonadectomy: a committee opinion. Fertil Steril.

[7] Anderson R, Amant Frédéric, Braat Didi, D'Angelo Arianna, Chuva de Sousa Lopes Susana M, Demeestere Isabelle, Dwek Sandra, Frith Lucy, Lambertini Matteo, Maslin Caroline, Moura-Ramos Mariana, Nogueira Daniela, Rodriguez-Wallberg Kenny, Vermeulen Nathalie, ESHRE Guideline Group on Female Fertility Preservation (2020). ESHRE guideline: female fertility preservation. Hum Reprod Open.

[8] Suzuki N (2019). Clinical practice guidelines for fertility preservation in pediatric, adolescent, and young adults with cancer. Int J Clin Oncol.

[9] Harada Miyuki, Kimura Fuminori, Takai Yasushi, Nakajima Takeshi, Ushijima Kimio, Kobayashi Hiroaki, Satoh Toyomi, Tozawa Akiko, Sugimoto Kohei, Saji Shigehira, Shimizu Chikako, Akiyama Kyoko, Bando Hiroko, Kuwahara Akira, Furui Tatsuro, Okada Hiroshi, Kawai Koji, Shinohara Nobuo, Nagao Koichi, Kitajima Michio, Suenobu Souichi, Soejima Toshinori, Miyachi Mitsuru, Miyoshi Yoko, Yoneda Akihiro, Horie Akihito, Ishida Yasushi, Usui Noriko, Kanda Yoshinobu, Fujii Nobuharu, Endo Makoto, Nakayama Robert, Hoshi Manabu, Yonemoto Tsukasa, Kiyotani Chikako, Okita Natsuko, Baba Eishi, Muto Manabu, Kikuchi Iwaho, Morishige Ken-Ichirou, Tsugawa Koichiro, Nishiyama Hiroyuki, Hosoi Hajime, Tanimoto Mitsune, Kawai Akira, Sugiyama Kazuhiko, Boku Narikazu, Yonemura Masato, Hayashi Naoko, Aoki Daisuke, Osuga Yutaka, Suzuki Nao (2022). Japan Society of Clinical Oncology Clinical Practice Guidelines 2017 for fertility preservation in childhood, adolescent, and young adult cancer patients: part 1. Int J Clin Oncol.

[10] Mulder RL, Font-Gonzalez A, Green DM, Loeffen EAH, Hudson MM, Loonen J, Yu R, Ginsberg JP, Mitchell RT, Byrne J, Skinner R, Anazodo A, Constine LS, de Vries A, Jahnukainen K, Lorenzo A, Meissner A, Nahata L, Dinkelman-Smit M, Tournaye H, Haupt R, van den Heuvel-Eibrink MM, van Santen HM, van Pelt AMM, Dirksen U, den Hartogh J, van Dulmen-den Broeder E, Wallace WH, Levine J, Tissing WJE, Kremer LCM, Kenney LB, van de Wetering MD, Berger C, Diesch T, Giwercman A, Grabow D, Gracia C, Hunter SE, Inthorn J, Kaatsch P, Kelvin JF, Klosky JL, Laven JSE, Lockart BA, Neggers SJ, Paul NW, Peate M, Phillips B, Quinn GP, Reed DR, Tinner EME, van den Berg M, Verhaak C (2021). Fertility preservation for male patients with childhood, adolescent, and young adult cancer: recommendations from the PanCareLIFE Consortium and the International Late Effects of Childhood Cancer Guideline Harmonization Group. Lancet Oncol.

[11] Mulder RL, Font-Gonzalez A, Hudson MM, van Santen HM, Loeffen EAH, Burns KC, Quinn GP, van Dulmen-den Broeder E, Byrne J, Haupt R, Wallace WH, van den Heuvel-Eibrink MM, Anazodo A, Anderson RA, Barnbrock A, Beck JD, Bos AME, Demeestere I, Denzer C, Di Iorgi N, Hoefgen HR, Kebudi R, Lambalk C, Langer T, Meacham LR, Rodriguez-Wallberg K, Stern C, Stutz-Grunder E, van Dorp W, Veening M, Veldkamp S, van der Meulen E, Constine LS, Kenney LB, van de Wetering MD, Kremer LCM, Levine J, Tissing WJE, Berger C, Diesch T, Dirksen U, Ginsberg J, Giwercman A, Grabow D, Gracia C, Hunter SE, Inthorn J, Kaatsch P, Kelvin JF, Klosky JL, Laven JSE, Lockart BA, Neggers SJ, Paul NW, Peate M, Phillips B, Reed DR, Tinner EME, van den Berg M, Verhaak C (2021). Fertility preservation for female patients with childhood, adolescent, and young adult cancer: recommendations from the PanCareLIFE Consortium and the International Late Effects of Childhood Cancer Guideline Harmonization Group. Lancet Oncol.

[12] Winship AL, Alesi LR, Sant S, Stringer JM, Cantavenera A, Hegarty T, Requesens CL, Liew SH, Sarma U, Griffiths MJ, Zerafa N, Fox SB, Brown E, Caramia F, Zareie P, La Gruta NL, Phillips K, Strasser A, Loi S, Hutt KJ (2022). Checkpoint inhibitor immunotherapy diminishes oocyte number and quality in mice. Nat Cancer.

[13] Ussher JM, Perz J (2019). Infertility-related distress following cancer for women and men: a mixed method study. Psychooncology.

[14] FertiPROTEKT.

[15] SGRM.

[16] REDCap.

[17] Cooper Trevor G, Noonan E, von Eckardstein Sigrid, Auger Jacques, Baker H W Gordon, Behre Hermann M, Haugen Trine B, Kruger Thinus, Wang Christina, Mbizvo Michael T, Vogelsong Kirsten M (2010). World Health Organization reference values for human semen characteristics. Hum Reprod Update.

[18] Segawa T, Omi K, Watanabe Y, Sone Y, Handa M, Kuroda M, Miyauchi O, Osada H, Teramoto S (2019). Age-specific values of Access anti-Müllerian hormone immunoassay carried out on Japanese patients with infertility: a retrospective large-scale study. BMC Womens Health.

[19] Zentrum für Krebsregisterdaten.

[20] FertiTOX.

[21] Dezellus A, Barriere P, Campone M, Lemanski C, Vanlemmens L, Mignot L, Delozier T, Levy C, Bendavid C, Debled M, Bachelot T, Jouannaud C, Loustalot C, Mouret-Reynier M, Gallais-Umbert A, Masson D, Freour T (2017). Prospective evaluation of serum anti-Müllerian hormone dynamics in 250 women of reproductive age treated with chemotherapy for breast cancer. Eur J Cancer.

[22] Perdrix A, Saint-Ghislain M, Degremont M, David M, Khaznadar Z, Loeb A, Leheurteur M, Di Fiore F, Clatot F (2017). Influence of adjuvant chemotherapy on anti-Müllerian hormone in women below 35 years treated for early breast cancer. Reprod Biomed Online.

[23] Trapp E, Steidl J, Rack B, Kupka M, Andergassen U, Jückstock J, Kurt A, Vilsmaier T, de Gregorio A, de Gregorio N, Tzschaschel M, Lato C, Polasik A, Tesch H, Schneeweiss A, Beckmann M, Fasching P, Janni W, Müller V (2017). Anti-Müllerian hormone (AMH) levels in premenopausal breast cancer patients treated with taxane-based adjuvant chemotherapy - A translational research project of the SUCCESS A study. Breast.

[24] Zhou B, Kwan B, Desai MJ, Nalawade V, Ruddy KJ, Nathan PC, Henk HJ, Murphy JD, Whitcomb BW, Su HI (2022). Long-term antimüllerian hormone patterns differ by cancer treatment exposures in young breast cancer survivors. Fertil Steril.

