# Supplementary material for: Evaluation of the Gonadotoxicity of Cancer Therapies to Improve Counseling of Patients About Fertility and Fertility Preservation Measures: Protocol for a Retrospective Systematic Data Analysis and a Prospective Cohort Study
Source: JMIR Res Protoc. 2024 Mar 20;13:e51145. doi: 10.2196/51145 (PMC10993117; doi:10.2196/51145)
Supplement: Multimedia Appendix 1 [file resprot_v13i1e51145_app1.docx]

**Multimedia Appendix 1**

**Centers participating the prospective cohort study (in alphabetical order)**

Germany

- Aachen: Universitätsklinikum RWTH Aachen, Frauenklinik für Gynäkologische Endokrinologie und Reproduktionsmedizin - Dr. med. Nele Freerksen-Kirschner
- Berlin: Charité Universitätsmedizin Berlin, Charité, Centrum für Frauen-, Kinder- und Jugendmedizin mit Perinatalzentrum und Humangenetik, Klinik für Gynäkologie mit Zentrum für onkologische Chirurgie - Prof. Dr. med. Dr. h.c. Jalid Sehouli, Dr. med. Judith Altmann
- Berlin: Fertility Center Berlin - Prof. Dr. med. Heribert Kentenich, Dr. med. Andreas Tandler-Schneider
- Berlin: Kinderwunschzentrum an der Gedächtniskirche Berlin - Dr. med. Matthias Bloechle, Dr. med. Silke Marr
- Bielefeld: Bielefeld Fertility Center - Dr. med. Karl Völklein
- Bonn: Universitätsklinikum Bonn, Venuskind am UKB, Kinderwunschzentrum - Prof. Dr. med. Nicole Sänger, Dr. med. Julia John
- Bremen: Klinikum Bremen Mitte, Klinik für Gynäkologie - Dr. med. Mustafa Aydogdu
- Dortmund: MVZ Kinderwunschzentrum Dortmund GmbH - Prof. Dr. med. Stefan Dieterle
- Dresden: Universitätsklinikum Carl Gustav Carus an der Technischen Universität Dresden, Klinik und Poliklinik für Frauenheilkunde und Geburtshilfe - Dr. med. Maren Goeckenjan-Festag
- Düsseldorf: Universitätsklinikum Düsseldorf, UniKid Universitäres Interdisziplinäres Kinderwunschzentrum Düsseldorf - Prof. Dr. med. Jan-Steffen Krüssel
- Erlangen: Uniklinikum Erlangen, Frauenklinik - Prof. Dr. rer. nat. Ralf Dittrich
- Frankfurt: Re-Pro Gyn Universitätsklinikum Frankfurt - Rahila Nuriyeva, Aynura Abbasova-Semiz
- Freiburg: Universitätsklinikum Freiburg, Klinik für Frauenheilkunde - Dr. med. Philipp Wiehle, Dr. med. Jasmin Asberger
- Gießen: UKGM Universitätsklinikum Gießen und Marburg GmbH, Standort Gießen, Klinik und Poliklinik für Urologie, Kinderurologie und Andrologie - Prof. Dr. med. Hans-Christian Schuppe, Prof. Dr. med. Adrian Pilatz, Prof. Dr. med. Florian Wagenlehner
- Gießen: UKGM Universitätsklinikum Gießen und Marburg GmbH, Standort Marburg, Klinik für Frauenheilkunde und Geburtshilfe - Prof. (apl.) Dr. med. Volker Ziller
- Göttingen: UMG Universitätsmedizin Göttingen, Klinik für Gynäkologie und Geburtshilfe - Priv.-Doz. Dr. med. Gerd J. Bauerschmitz PhD
- Greifswald: Universitätsmedizin Greifswald, Klinik und Poliklinik für Frauenheilkunde und Geburtshilfe - Univ.-Prof. Dr. med. Marek Zygmunt
- Halle: Universitätsklinikum Halle (Saale), Zentrum für Reproduktionsmedizin und Andrologie (ZRA) - Prof. Dr. med. Hermann M. Behre
- Hamburg: amedes experts hamburg Facharzt-Zentrum für Kinderwunsch, Pränatale Medizin, Endokrinologie und Osteologie - Prof. Dr. med. Frank Nawroth
- Hannover: MHH Medizinische Hochschule Hannover, Klinik für Frauenheilkunde und Geburtshilfe - Prof. Dr. med. Cordula Schippert, Prof. Dr. med. Frauke von Versen-Höynck
- Heidelberg: Universitätsklinikum Heidelberg - Prof. Dr. med. Ariane Germeyer
- Heidelberg: Universitätsklinikum Heidelberg, National Center for Tumor Diseases (NCT) Heidelberg - Prof. Dr. med. Carsten Müller-Tidow und Prof. Dr. med. Andreas Schneeweiss
- Hildesheim: Zentrum für Reproduktionsmedizin und Humangenetik Hildesheim - Dr. med. Jan-Simon Lanowski
- Karlsruhe: Städtisches Klinikum Karlsruhe, Frauenklinik - Prof. Dr. med. Andreas Müller
- Kassel: MVZ Medizinisches Versorgungszentrum für Reproduktionsmedizin am Klinikum Kassel - Dr. med. Marc Janos Willi, Dr. med. Oswald Schmidt
- Kiel: Universitäres Kinderwunschzentrum Kiel Lübeck Manhagen - PD Dr. med. Sören von Otte
- Köln: MVZ PAN-Institut Köln - Dr. med. Dipl.-Biol. Irene Pütz
- Köln: Universitätsklinikum Köln (AöR), Klinik und Poliklinik für Frauenheilkunde und Geburtshilfe - Prof. Dr. med. Gohar Rahimi
- Leipzig: Universitätsklinikum Leipzig, Klinik und Poliklinik für Dermatologie, Venerologie und Allergologie - Prof. Dr. med. Sonja Grunewald
- Lübeck: Universitätsklinikum Schleswig-Holstein, Sektion für gynäkologische Endokrinologie und Reproduktionsmedizin - Prof. Dr. med. univ. Georg Griesinger, MSc
- Magdeburg: Universitätsklinikum Magdeburg A.ö.R., Universitätsklinik für Frauenheilkunde, Geburtshilfe und Reproduktionsmedizin - Carina Strecker
- Mainz: Universitätsmedizin der Johannes Gutenberg-Universität Mainz, Universitäts-Kinderwunschzentrum und Ambulanz für Gynäkologische Endokrinologie - Prof. Dr. med. Christine Skala, Dr. med. Susanne Theis
- München: Kinderwunschzentrum A.R.T. Bogenhausen - Dr. med. Stephanie Ziehr
- München: LMU Klinikum der Universität München, Klinik und Poliklinik für Frauenheilkunde und Geburtshilfe, LMU Hormon- und Kinderwunschzentrum - Univ.- Prof. Dr. med. Christian J. Thaler, Prof. Dr. med. Nina Rogenhofer
- München: TUM Klinikum rechts der Isar, Technische Universität München, Klinik und Poliklinik für Frauenheilkunde - Prof. Dr. med. Vanadin Seifert-Klauss, Univ.-Prof. Dr. med. Marion Kiechle
- Münster: UKM Universitätsklinikum, Münster Kinderwunschzentrum - Univ.-Prof. Dr. med. Hermann M. Behre
- Münster: UKM Universitätsklinikum, Münster Centrum für Reproduktionsmedizin und Andrologie - Priv. Doz. Dr. rer. nat. Verena Nordhoff, Univ.-Prof. Dr. rer. nat. Stefan Schlatt, Prof. Dr. med. Sabine Kliesch
- Oldenburg: Tagesklinik Oldenburg - Dr. med. Jörg Hennefründ (Dipl. Biologin Gabriele Remek)
- Regensburg: Profertilita Zentrum für Fruchtbarkeitsmedizin - Dr. med. Karla Gisch-Pratsch MSc, Dr. med. Christine Reißmann
- Rostock: Praxis für Fertilität MVZ Gmbh Rostock - inviTRA - Priv. Doz. Dr. med. Heiner Müller, Dr. med. Anne Koenen
- Saarland: Universitätsklinikum des Saarlandes, Klinik für Frauenheilkunde, Geburtshilfe und Reproduktionsmedizin Homburg (Saar) - Dr. med. Simona L. Baus
- Tübingen: Universitäts-Frauenklinik, Department für Frauengesundheit Tübingen - Priv. Doz. Dr. med. Melanie Henes
- Ulm: Universitätsklinikum Ulm, Klinik für Frauenheilkunde und Geburtshilfe, Kinderwunschzentrum UniFee - Prof. Dr. med. Katharina Hancke
- Wiesbaden: MVZ Kinderwunsch am Welfenhof - Karin Schilberz
- Würzburg: Kinderwunschzentrum der Universitätsklinik Würzburg, Zentrum für gynäkologische Endokrinologie und Reproduktionsmedizin (ZERM) - Dr. med. Michael Schwab

Switzerland

- Baden-Dättwil: Baden Täfernhof - Dr. med. Mischa Schneider
- Basel: Regio Basel - Dr. med. Erika Ocon
- Basel: Universitätsspital Basel - Dr. med. Ursula Gobrecht-Keller
- Bern: Inselspital - Prof. Dr. med. Michael von Wolff
- Bern: Lindenhofspital - Dr. med. Elisabeth Berger-Menz
- Biel: CARE - Dr. med. Susanna Crazzolara
- Chur: Kantonsspital Graubünden, Fontana - Dr. med. Naomi Ventura
- Fribourg: HFR - PD Dr. med. Dorothea Wunder
- Genève: HUG – Dr. Federico Del Vento
- Küsnacht: GYNE INVITRO - Dr. med. Michael Singer
- Lausanne: CHUV - Dr. Anna Surbone
- Lausanne: CPMA - Dr. med. Nicolas Vulliemoz
- Locarno: Centro Cantonale di Fertilità - Dr. med. Alessandro Santi
- Luzern: Kantonsspital Luzern - PD Dr. med. Alexandra Kohl Schwartz
- Luzern: Klinik St. Anna - Dr. med. Sabine Steimann
- Olten: Fertisuisse - PD Dr. med. Gideon Sartorius
- St. Gallen: Yuna - Dr. med. Vera Hungerbühler
- Winterthur: Admira, Winterthur - Dr. med. Monika Fäh
- Zürich: 360 Grad, Zürich - Dr. med. Florian Götze
- Zürich: GYN-A.R.T. AG - Moritz Suerdieck
- Zürich: OVA - IVF Clinic - Dr. med. Peter Fehr

Austria

- Graz: Medical University Graz - Dr. Marion Neumayer
- Innsbruck: Medical University of Innsbruck - PD Dr. med. Bettina Böttcher MA
- Linz: Johannes Kepler University Linz - PD Dr. Omar Josef Shebl
- Salzburg: Private Medical University, Salzburg - Dr. Katharina Winkler- Crepaz
- Wien: Medical University of Vienna - PD Dr. med. univ. Julian Marschalek
- Wien: Saint Anna Children's Hospital – Univ.-Doz. Dr. Michael N. Dworzak
