# Supplementary material for: Evaluation of the Gonadotoxicity of Cancer Therapies to Improve Counseling of Patients About Fertility and Fertility Preservation Measures: Protocol for a Retrospective Systematic Data Analysis and a Prospective Cohort Study
Source: JMIR Res Protoc. 2024 Mar 20;13:e51145. doi: 10.2196/51145 (PMC10993117; doi:10.2196/51145)
Supplement: Multimedia Appendix 3 [file resprot_v13i1e51145_app3.pdf]

FertiTOX
Fertility after gonadotoxic treatments

Basic Patient Information

♂

Record ID	

Automatically generated by REDCap.

Study centre

Name of the study centre		*See **annexed list** with the cities listed alphabetically for Austria, Germany and Switzerland.*

if ➡ NOT LISTED	Country	
	City	
	Centre	

Centre specific ID code of the patient	*Optional.*	
Date of first consultation		*Min: 01-11-2023*<br>*Max: 31-12-2039*

Diagnosis

Disease	*Preliminary diagnosis, for which the fertility counselling is performed.*	*See **annexed file** with the disease list.*

if ➡ NOT LISTED	

Date of diagnosis		*Min: 01-01-2020*<br>*Max: 31-12-2039*

Annex → Fertility Centres

♂

AUSTRIA

- ☐ **Graz** – *Med Uni*
- ☐ **Innsbruck** – *Medical University*
- ☐ **Linz** – *JKU*
- ☐ **Salzburg** – *PMU*
- ☐ **Wien** – *MedUni*
- ☐ **Wien** – *St. Anna Kinderspital*

GERMANY

- ☐ **Aachen** – *RWTH*
- ☐ **Berlin** – *"an der Gedächtniskirche"*
- ☐ **Berlin** – *Charité*
- ☐ **Berlin** – *Fertility Centre*
- ☐ **Bielefeld** – *Fertility Centre*
- ☐ **Bonn** – *Venuskind am UKB*
- ☐ **Bremen** – *Mitte Clinic*
- ☐ **Dortmund** – *MVZ*
- ☐ **Dresden** – *Carl Gustav Carus*
- ☐ **Düsseldorf** – *UniKid*
- ☐ **Erlangen** – *University Hospital*
- ☐ **Frankfurt** – *Re-Pro Gyn*
- ☐ **Freiburg** – *University Hospital*
- ☐ **Gießen** – *UKGM (Andrology)*
- ☐ **Göttingen** – *UMG*
- ☐ **Greifswald** – *University Hospital*
- ☐ **Halle (Saale)** – *ZRA*
- ☐ **Hamburg** – *"amedes experts"*
- ☐ **Hannover** – *MHH*
- ☐ **Heidelberg** – *NCT*
- ☐ **Heidelberg** – *UKHD*
- ☐ **Hildesheim** – *Fertility Centre*
- ☐ **Karlsruhe** – *City Clinic*
- ☐ **Kassel** – *MVZ*
- ☐ **Kiel** – *UKSH Lübeck Manhagen*
- ☐ **Köln** – *MVZ PAN-Institute*
- ☐ **Köln** – *University Hospital*
- ☐ **Leipzig** – *University Hospital*
- ☐ **Lübeck** – *UKSH Schleswig-Holstein*
- ☐ **Magdeburg** – *OVGU*
- ☐ **Mainz** – *JGU*
- ☐ **Marburg** – *UKGM (Gynaecology)*
- ☐ **München** – *ART Bogenhausen*
- ☐ **München** – *LMU*
- ☐ **München** – *TUM*
- ☐ **Münster** – *UKM (Andrology)*
- ☐ **Münster** – *UKM (Fertility Centre)*
- ☐ **Oldenburg** – *Fertility Centre*
- ☐ **Regensburg** – *Profertilita*
- ☐ **Rostock** – *Fertility Centre*
- ☐ **Saarland** – *UKS*
- ☐ **Tübingen** – *University Hospital*
- ☐ **Ulm** – *UULM*
- ☐ **Wiesbaden** – *TFP*
- ☐ **Würzburg** – *University Hospital*

SWITZERLAND

- ☐ **Baden** – *Fertility Centre*
- ☐ **Basel** – *Fertility Centre*
- ☐ **Basel** – *University Hospital*
- ☐ **Bern** – *Fertility Centre (Lindenhofspital)*
- ☐ **Bern** – *Inselspital*
- ☐ **Biel** – *CARE*
- ☐ **Chur** – *Fontana*
- ☐ **Fribourg** – *HFR*
- ☐ **Genève** – *HUG*
- ☐ **Lausanne** – *CHUV*
- ☐ **Lausanne** – *CPMA*
- ☐ **Locarno** – *La Carità*
- ☐ **Luzern** – *LUKS*
- ☐ **Luzern** – *St. Anna*
- ☐ **Olten** – *fertisuisse*
- ☐ **St. Gallen** – *YUNA*
- ☐ **Winterthur** – *Admira*
- ☐ **Zürich** – *360 Grad*
- ☐ **Zürich** – *GYN-A.R.T.*
- ☐ **Zürich** – *Gyné invitro*
- ☐ **Zürich** – *OVA IVF*
- ☐ **Zürich** – *USZ*

Annex ➜ Disease List ♂

International Statistical Classification of Diseases and Related Health Problems 10th Revision (ICD-10)-WHO, Version for 2019-covid-expanded.

A
Agranulocytosis (D70)
Anal carcinoma (C21)
Anaemia - Aplastic (D60 – D61)
Anaemia - Fanconi (D61.0)
Angiosarcoma (C22.3)

B
Blasenmole ➜ **»Trophoblastic disease«**
Brain cancer:
- *Astrocytoma (C71.9)*
- *Ependymoma (C71.9)*
- *Glioblastoma (C71.9)*
- *Oligodendroglioma (C71.9)*
- *Medulloblastoma (C71.6)*
- *other (C71)*

Breast cancer:
- *benign BRCA positive (Z15.01)*
- *hormone receptor positive (Z17.0)*
- *hormone receptor negative (Z17.1)*
- *hormone receptor status not known (Z17)*
- *other (C50)*

Burkitt lymphoma (C83.7)

C
Cervix uteri carcinoma (C53)
Chondrosarcoma (C41.9)
Colitis ulcerosa ➜ **»Ulcerative colitis«**
Colon carcinoma (C18)
Crohn disease (K50)

D
Dermatomyositis (M33)

E
Endometrial carcinoma (C54)
Endometriosis (N80)
Ewing sarcoma (C40-C41)

F
Fibrosarcoma (C49.9)
Fragile X syndrome (Q99.2)

G
Galactosemia (E74.2)
Germ cell tumour - extragonadal (ICD-O-3 M906-909)

H
Hodgkin lymphoma (C81)

I
Immune thrombocytopenia (D69)

K
Keimzelltumor ➜ **»Germ cell tumour«**
Kidney cancer (C64)

L
Leiomyosarcoma NOS (ICD-O-3 M8890/3)
Leukaemia:
- *Leukaemia lymphoid - acute lymphoblastic (C91.0)*
- *Leukaemia lymphoid - chronic lymphocytic (C91)*
- *Leukaemia myeloid - acute (C92)*
- *Leukaemia myeloid - chronic (C92)*
- *Leukaemia - other forms (C91 - C95)*

Liposarcoma (C49.9)
Liver cancer (C22)
Lung cancer (C34)
Lupus erythematosus (L93)

M
Malignant fibrous histiocytoma ➜ **»Undifferentiated pleomorphic sarcoma«**
Malignant nerve sheath tumour (C47.9)
Melanoma (C43)
Mesothelioma (C45)
Mixed connective tissue disease (M35.1)
Morbus Crohn ➜ **»Crohn disease«**
Myelodysplastic syndrome (D46)
Multiple sclerosis (G35)
Myositis (M60)

N
Nasopharyngeal carcinoma (C11)
Nebennierenkrebs ➜ **»Adrenocortical carcinoma«**
Nephritis / Glomerulonephritis (N05)
Nephroblastoma ➜ **»Wilms' tumour«**
Non-Hodgkin lymphoma (C82 – C88)

O
Osteosarcoma (C41.9)
Ovary (C56):
- *borderline malignancy*
- *teratoma benign*
- *teratoma malignant*
- *other*

P
Pancreatic cancer (C25)
Placenta cancer (C58.9; D39.2) ➜ **»Trophoblastic disease«**
Pleomorphic undifferentiated sarcoma ➜ **»Undifferentiated pleomorphic sarcoma«**
Polyarteritis nodosa (M30)
Polychondritis (M94.8)
Polymyositis (M33)
Premature ovarian insufficiency (E28.3)
Prostate cancer (C61)

R
Rectal cancer (C20)
Rectosigmoid junction cancer (C19)
Renal cell carcinoma ➜ **»Kidney cancer«**
Rhabdomyosarcoma (C49.9)
Rheumatoid arthritis (M05 – M06, M08)

S
Schilddrüsenkrebs ➜ **»Thyroid cancer«**
Sharp syndrome ➜ **»Mixed connective tissue disease«**
Sickle cell disease (D57)
Sigmakarzinom ➜ **»Colon carcinoma«**
Sjögren (Sicca) syndrome (M35)
Stomach cancer (C16)
Synovial sarcoma (C49.9)
Systemic sclerosis (including: Scleroderma) (M34)

T
Takayasu arteritis (Aortic arch syndrome) (M31.4)
Testicular cancer (C62):
- *seminoma*
- *non seminomatous germ cell tumour - teratoma*
- *non seminomatous germ cell tumour - embryonal carcinoma*
- *non seminomatous germ cell tumour - yolk sac carcinoma*
- *stromal tumour - Leydig cell tumour*
- *stromal tumour - Sertoli cell tumour*
- *other histological types*

Thalassemia (D56)
Thyroid cancer (C73)
Transgender (F64)
Trophoblastic disease (O01.9)
Turner syndrome (Q96)

U
Ulcerative colitis (K51)
Undifferentiated pleomorphic sarcoma (C49)

V
Vasculitis limited to skin (L95)
Vulva carcinoma (C51)

W
Wegener granulomatosis (M31.3)
Wilms' tumour (Kidney cancer in children) (C64)

?
DISEASE NOT LISTED

Record ID	

Basic information

Sex	☐ Male<br>☐ Female	
Year of birth		*Integer, suggested range: 1973 - 2009*
Age at inclusion	*years*	***Automatic calculation** of rounded number of years in REDCap.*
Height	*cm*	*Integer, suggested range: 110 - 210*
Weight	*kg*	*Integer, suggested range: 30 - 250*
BMI	*kg/m²*	***Automatic calculation** of BMI in REDCap.*
Smoking status	☐ Yes<br>☐ No	
Partnership	☐ Yes<br>☐ No	

Record ID	

Fertility

How many biological children does the patient have?

- ☐ 0
- ☐ 1 ➡ Age of 1st child ____ years
- ☐ 2 ➡ Age of 2nd child ____ years
- ☐ > 2 ➡ Age of 3rd child ____ years

Integer, suggested range: 0 - 25

Infertility

Previous infertility condition in patient and/or partner

- ☐ Yes

  if YES ➡ What was the cause of infertility?
  - ☐ Male
  - ☐ Female
  - ☐ Mixt
  - ☐ Idiopathic
  - ☐ Other

  ➡ if OTHER, please specify

  if YES ➡ What type of treatment for infertility was received?
  - ☐ Timed intercourse
  - ☐ Ovarian stimulation and timed intercourse
  - ☐ Intrauterine insemination
  - ☐ IVF / ICSI
  - ☐ Other

  ➡ if OTHER, please specify

- ☐ No

Fertility Preservation Measures ♂

Record ID ______

Sperm

Cryopreservation of sperm before gonadotoxic treatment?

☐ Yes

if YES ➡ Date of sperm cryopreservation ______ *Min: 01-11-2023 Max: 31-12-2039*

if YES ➡ Number of ejaculates
☐ 1
☐ 2
☐ 3
☐ 4
☐ 5
☐ > 5

☐ No

Testicular tissue

Cryopreservation of testicular tissue before gonadotoxic treatment?

☐ Yes

if YES ➡ Date of testicular tissue cryopreservation ______ *Min: 01-11-2023 Max: 31-12-2039*

if YES ➡ Complications during or just after the process of testicular tissue removal
☐ None
☐ Bleeding
☐ Infection
☐ Other
Several items can be chosen.

if OTHER ➡ please specify ______

☐ No

End Study Block Basic

Did you fill in all basic information and fertility preservation measures?
☐ Yes
☐ No

Eligibility

♂

Record ID	

Eligibility

Informed Consent	Signed	☐ Yes	➡ Date of signed 'Informed Consent'	*Min: 01-11-2023 Max: 31-12-2039*
		☐ No		

Rough estimated date	Expected end of gonadotoxic treatment affecting the ovaries / testicles	*Please add a rough estimate of the date in order* ***to schedule*** *the posttreatment consultation. Min: 01-11-2023 Max: 31-12-2039*

Inclusion Criteria	1	Patients with cancer or with benign reasons undergoing chemotherapy and/or radiotherapy of the pelvis (females) and the testicles (males) and/or immune therapy	☐ Yes ☐ No
	2	Willing to participate	☐ Yes ☐ No
	3	▪ Austria: 14-50 years old (adolescents and adults)<br>▪ Germany: 18-50 years old<br>▪ Switzerland: 14-50 years old (adolescents and adults)	☐ Yes ☐ No
	4	Serum hormone analysis before gonadotoxic therapy (females) or serum hormone analysis and sperm analysis before gonadotoxic therapy (males)	☐ Yes ☐ No

Exclusion Criteria	1	Missing consent	☐ Yes ☐ No
	2	Language barrier	☐ Yes ☐ No

Eligibility Determination	☐ Patient is **ELIGIBLE**<br>☐ Patient does **NOT** meet eligibility criteria<br>☐ Eligibility criteria are **INCOMPLETE**

Automatically generated by REDCap.

Pretreatment Fertility Status

Record ID	

Blood parameters

Field	Entry	Notes
Date of blood test		Min: 01-11-2023<br>Max: 31-12-2039
	Time of blood collection: ideally in the morning, between 8-12 am.	
Serum total testosterone concentration		*Number (one decimal place), suggested range: 0.0 - 2000.0*
Unit of the serum total testosterone value	☐ nmol/L<br>☐ ng/dL<br>☐ microgram/L	

Sperm parameters

Field	Entry	Notes
Date of sperm analysis (first ejaculate)		*Min: 01-11-2023*<br>*Max: 31-12-2039*
Total sperm count in ejaculate	10^6	*Number (one decimal place), suggested range: 0.0 - 928.0*
Sperm concentration	10^6/mL	*Number (one decimal place), suggested range: 0.0 - 300.0*
Total sperm progressive motility (WHO A+B)	%	*Integer, suggested range: 5 - 85*
Proportion of sperm with normal morphology	%<br>*If analysed in first ejaculate*	*Integer, suggested range: 0 - 30*

Legend:
A = rapid progressive
B = slow progressive
C = non-progressive
D = immotility
