# Supplementary material for: Evaluation of the Gonadotoxicity of Cancer Therapies to Improve Counseling of Patients About Fertility and Fertility Preservation Measures: Protocol for a Retrospective Systematic Data Analysis and a Prospective Cohort Study
Source: JMIR Res Protoc. 2024 Mar 20;13:e51145. doi: 10.2196/51145 (PMC10993117; doi:10.2196/51145)
Supplement: Multimedia Appendix 4 [file resprot_v13i1e51145_app4.pdf]

FertiTOX
Fertility after gonadotoxic treatments

Posttreatment 12-15 months

♀

Record ID | |

Visit

**Date of posttreatment visit at 12-15 months after the end of gonadotoxic treament**		*Min: 01-11-2023*<br>*Max: 31-12-2039*

Gonadotoxic treatment

**Disease**	*Final diagnosis, for which the fertility counselling is performed.*	*See **annexed list** with the disease list.*
**if ➡ NOT LISTED**		
**Start of gonadotoxic treatment**		*Min: 01-01-2020*<br>*Max: 31-12-2039*
**End of gonadotoxic treatment**		*Min: 01-01-2020*<br>*Max: 31-12-2039*
**Duration of gonadotoxic treatment**	*Months*	***Automatic calculation** of rounded number of months in REDCap.*
**Treatment protocol**<br>*(To be added by the organizers of the study.)*		*Numbered list from 1 to 99.*
**Have ovaries been removed as part of cancer therapy?**	▢ Yes<br>▢ No	
**if YES ➡ How many ovaries?**	▢ ½ ovary<br>▢ 1 ovary<br>▢ 2 ovaries	

FertiTOX
Fertility after gonadotoxic treatments

Posttreatment 12-15 months ♀

Record ID	

A
- *Agranulocytosis (D70)*
- *Anal carcinoma (C21)*
- *Anaemia - Aplastic (D60 – D61)*
- *Anaemia - Fanconi (D61.0)*
- *Angiosarcoma (C22.3)*

B
- *Blasenmole* ➜ **»Trophoblastic disease«**
- **Brain cancer:**
  - *Astrocytoma (C71.9)*
  - *Ependymoma (C71.9)*
  - *Glioblastoma (C71.9)*
  - *Oligodendroglioma (C71.9)*
  - *Medulloblastoma (C71.6)*
  - *other (C71)*
- **Breast cancer:**
  - *benign BRCA positive (Z15.01)*
  - *hormone receptor positive (Z17.0)*
  - *hormone receptor negative (Z17.1)*
  - *hormone receptor status not known (Z17)*
  - *other (C50)*
- *Burkitt lymphoma (C83.7)*

C
- *Cervix uteri carcinoma (C53)*
- *Chondrosarcoma (C41.9)*
- *Colitis ulcerosa* ➜ **»Ulcerative colitis«**
- *Colon carcinoma (C18)*
- *Crohn disease (K50)*

D
- *Dermatomyositis (M33)*

E
- *Endometrial carcinoma (C54)*
- *Endometriosis (N80)*
- *Ewing sarcoma (C40-C41)*

F
- *Fibrosarcoma (C49.9)*
- *Fragile X syndrome (Q99.2)*

G
- *Galactosemia (E74.2)*
- *Germ cell tumour - extragonadal (ICD-O-3 M906-909)*

H
- *Hodgkin lymphoma (C81)*

I
- *Immune thrombocytopenia (D69)*

K
- *Keimzelltumor* ➜ **»Germ cell tumour«**
- *Kidney cancer (C64)*

L
- *Leiomyosarcoma NOS (ICD-O-3 M8890/3)*
- **Leukaemia:**
  - *Leukaemia lymphoid - acute lymphoblastic (C91.0)*
  - *Leukaemia lymphoid - chronic lymphocytic (C91)*
  - *Leukaemia myeloid - acute (C92)*
  - *Leukaemia myeloid - chronic (C92)*
  - *Leukaemia - other forms (C91 - C95)*
- *Liposarcoma (C49.9)*
- *Liver cancer (C22)*
- *Lung cancer (C34)*
- *Lupus erythematosus (L93)*

M
- *Malignant fibrous histiocytoma* ➜ **»Undifferentiated pleomorphic sarcoma«**
- *Malignant nerve sheath tumour (C47.9)*
- *Melanoma (C43)*
- *Mesothelioma (C45)*
- *Mixed connective tissue disease (M35.1)*
- *Morbus Crohn* ➜ **»Crohn disease«**
- *Myelodysplastic syndrome (D46)*
- *Multiple sclerosis (G35)*
- *Myositis (M60)*

N
- *Nasopharyngeal carcinoma (C11)*
- *Nebennierenkrebs* ➜ **»Adrenocortical carcinoma«**
- *Nephritis / Glomerulonephritis (N05)*
- *Nephroblastoma* ➜ **»Wilms' tumour«**
- *Non-Hodgkin lymphoma (C82 – C88)*

O
- *Osteosarcoma (C41.9)*
- **Ovary (C56):**
  - *borderline malignancy*
  - *teratoma benign*
  - *teratoma malignant*
  - *other*

P
- *Pancreatic cancer (C25)*
- *Placenta cancer (C58.9; D39.2)* ➜ **»Trophoblastic disease«**
- *Pleomorphic undifferentiated sarcoma* ➜ **»Undifferentiated pleomorphic sarcoma«**
- *Polyarteritis nodosa (M30)*
- *Polychondritis (M94.8)*
- *Polymyositis (M33)*
- *Premature ovarian insufficiency (E28.3)*
- *Prostate cancer (C61)*

R
- *Rectal cancer (C20)*
- *Rectosigmoid junction cancer (C19)*
- *Renal cell carcinoma* ➜ **»Kidney cancer«**
- *Rhabdomyosarcoma (C49.9)*
- *Rheumatoid arthritis (M05 – M06, M08)*

S
- *Schilddrüsenkrebs* ➜ **»Thyroid cancer«**
- *Sharp syndrome* ➜ **»Mixed connective tissue disease«**
- *Sickle cell disease (D57)*
- *Sigmakarzinom* ➜ **»Colon carcinoma«**
- *Sjögren (Sicca) syndrome (M35)*
- *Stomach cancer (C16)*
- *Synovial sarcoma (C49.9)*
- *Systemic sclerosis (including: Scleroderma) (M34)*

T
- *Takayasu arteritis (Aortic arch syndrome) (M31.4)*
- **Testicular cancer (C62):**
  - *seminoma*
  - *non seminomatous germ cell tumour - teratoma*
  - *non seminomatous germ cell tumour - embryonal carcinoma*
  - *non seminomatous germ cell tumour - yolk sac carcinoma*
  - *stromal tumour - Leydig cell tumour*
  - *stromal tumour - Sertoli cell tumour*
  - *other histological types*
- *Thalassemia (D56)*
- *Thyroid cancer (C73)*
- *Transgender (F64)*
- *Trophoblastic disease (O01.9)*
- *Turner syndrome (Q96)*

U
- *Ulcerative colitis (K51)*
- *Undifferentiated pleomorphic sarcoma (C49)*

V
- *Vasculitis limited to skin (L95)*
- *Vulva carcinoma (C51)*

W
- *Wegener granulomatosis (M31.3)*
- *Wilms' tumour (Kidney cancer in children) (C64)*

? **DISEASE NOT LISTED**

Record ID	

Chemotherapy

☐ Yes

if YES ➡ Protocol name (such as BEACOPPesc, CHOP, FEC, AC, etc.), medications given, dosage per cycle, number of cycles

☐ No

Radiotherapy

☐ Yes

if YES ➡ Protocol name, radiation field and applied dosage in case of pelvic radiation

☐ No

Record ID	

Immumotherapy

☐ Yes

if YES ➡ Protocol name, type of antibodies or inhibitors, dosage plus any other relevant details

☐ No

Operation / Surgery

☐ Yes

if YES ➡ Protocol name plus any other relevant details

☐ No

Other type of gonadotoxic treatment

☐ Yes

if YES ➡ Protocol name plus any other relevant details

☐ No

Record ID	

Disease status

Disease status

- ☐ Absence of tumor/disease (complete remission)
- ☐ Some tumor mass/disease left (>50% reduction = partial remission)
- ☐ Substantial tumor mass/disease left (≤50% reduction = no remission)
- ☐ Relapse (reappearance of tumor/disease)

Any important cancer treatment-related events, such as: further therapies, a second cancer, other diseases, etc.

- ☐ Yes
- ☐ No

if YES ➡ Please specify

Contraception or hormone replacement therapy

What is your speciality?

- ☐ Gynaecology
- ☐ Oncology
- ☐ Other specialities

if GYNAECOLOGY ➡ Methods for contraception or HRT used

- ☐ FSH reducing contraceptives (combined oral, transdermal, vaginal contraceptives or three month intracutaneous injections, known as DPMA, "3-Monats-Spritze")
- ☐ Non-FSH reducing contraceptives (progesterone only, subcutaneous implants or any IUD)
- ☐ Hormone replacement therapy (HRT)
- ☐ None of the above

➡ if ONCOLOGY or OTHER SPECIALITIES

Is there any method of contraception or hormone replacement therapy (HRT)?

- ☐ Yes
- ☐ No

if YES ➡ Methods for contraception or HRT used

FertiTOX Fertility after gonadotoxic treatments

Posttreatment 12-15 months ♀

Record ID	

Blood parameters

Date of blood test		*Min: 01-11-2023*<br>*Max: 31-12-2039*
Serum AMH concentration		*Number (one decimal place), suggested range: 0.0 - 150.0*
Unit of the serum AMH value	☐ pmol/L<br>☐ ng/mL<br>☐ microgram/L	
Serum FSH concentration	*IU/L*	*Number (one decimal place), suggested range: 0.0 - 160.0*
Serum LH concentration	*IU/L*	*Number (one decimal place), suggested range: 0.0 - 100.0*
Serum E2 concentration		*Number (one decimal place), suggested range: 0.0 - 2000.0*
Unit of serum E2 value	☐ pmol/L<br>☐ ng/L<br>☐ pg/L	

Menstrual cycle parameters

How is the menstrual cycle?	☐ Regular (21-35 days)<br>☐ Regular (due to hormones, such as oral contraceptives and hormone replacement therapy)<br>☐ Irregular (oligomenorrhea, polymenorrhea, etc.)<br>☐ Amenorrhea = no or rare bleeding (due to premature ovarian insufficiency)<br>☐ Amenorrhea (due to other reasons, such as pregnancy, breastfeeding, hormone treatment, etc.)<br>In the last 3-6 months.
Day of menstrual cycle when blood test was taken	☐ Day 1-5<br>☐ After day 5<br>☐ Cannot be specified (amenorrhoea etc.)<br>☐ Unknown

Record ID	

Fertility preservation measures

Type of fertility preservation measure performed	☐ None<br>☐ GnRH analogues<br>☐ Oocyte freezing<br>☐ Zygote freezing<br>☐ Embryo freezing<br>☐ Ovarian tissue freezing<br>☐ Transposition of the ovaries<br>*Several items can be chosen.*

Patient satisfaction

Is the patient satisfied with the decision to have undergone fertility preservation measures or not?	☐ Yes<br>☐ No

How satisfied is the patient with the decision to undergo or not to undergo fertility preservation measures?											
	0	1	2	3	4	5	6	7	8	9	10

How satisfied is the patient with the fertility preservation counselling before the gonadotoxic treatment?											
	0	1	2	3	4	5	6	7	8	9	10

Slider labels:
***0** = not applicable (N/A)*
***1** = not satisfied at all*
***10** = very satisfied*

Pregnancy outcomes USING fertility preservation measures

Was frozen material used to achieve pregnancy?	☐ Yes	if YES ➡	Total number of pregnancies achieved after the gonadotoxic treatment using their own previous frozen material	☐ 0<br>☐ 1<br>☐ 2<br>☐ 3<br>☐ > 3
	☐ No			

1st Pregnancy		
	Frozen material	☐ Oocyte freezing<br>☐ Embryo freezing<br>☐ Ovarian tissue freezing<br>☐ Unknown
	Status	☐ Live birth<br>☐ Ongoing pregnancy<br>☐ Miscarriage - 1st trimester (< 14 weeks)<br>☐ Miscarriage - 2nd trimester (< 22 weeks)<br>☐ Stillbirth (≥ 22 weeks and weight ≥ 500 g)<br>☐ Induced abortion<br>☐ Ectopic pregnancy<br>☐ Other
	if OTHER ➡ please specify	

2nd Pregnancy		
	Frozen material	☐ Oocyte freezing<br>☐ Embryo freezing<br>☐ Ovarian tissue freezing<br>☐ Unknown
	Status	☐ Live birth<br>☐ Ongoing pregnancy<br>☐ Miscarriage - 1st trimester (< 14 weeks)<br>☐ Miscarriage - 2nd trimester (< 22 weeks)<br>☐ Stillbirth (≥ 22 weeks and weight ≥ 500 g)<br>☐ Induced abortion<br>☐ Ectopic pregnancy<br>☐ Other
	if OTHER ➡ please specify	

Record ID	

Pregnancy outcomes USING fertility preservation measures

3rd Pregnancy	Frozen material	☐ Oocyte freezing<br>☐ Embryo freezing<br>☐ Ovarian tissue freezing<br>☐ Unknown
	Status	☐ Live birth<br>☐ Ongoing pregnancy<br>☐ Miscarriage - 1st trimester (< 14 weeks)<br>☐ Miscarriage - 2nd trimester (< 22 weeks)<br>☐ Stillbirth (≥ 22 weeks and weight ≥ 500 g)<br>☐ Induced abortion<br>☐ Ectopic pregnancy<br>☐ Other
	if OTHER ➡ please specify	

4th Pregnancy	Frozen material	☐ Oocyte freezing<br>☐ Embryo freezing<br>☐ Ovarian tissue freezing<br>☐ Unknown
	Status	☐ Live birth<br>☐ Ongoing pregnancy<br>☐ Miscarriage - 1st trimester (< 14 weeks)<br>☐ Miscarriage - 2nd trimester (< 22 weeks)<br>☐ Stillbirth (≥ 22 weeks and weight ≥ 500 g)<br>☐ Induced abortion<br>☐ Ectopic pregnancy<br>☐ Other
	if OTHER ➡ please specify	

Posttreatment 12-15 months

♀

Record ID	

Pregnancy outcomes NOT USING their previous fertility preservation measures

Total number of pregnancies achieved after the gonadotoxic treatment NOT using their own previous frozen material	☐ 0<br>☐ 1<br>☐ 2<br>☐ 3<br>☐ > 3

0 Pregnancies	Has the patient tried to get pregnant after the end of gonadotoxic treatment?	☐ Yes<br>☐ No

1st Pregnancy	Method	☐ Natural conception (sexual intercourse)<br>☐ ART (Assisted Reproductive Technology)<br>☐ Unknown
	Status	☐ Live birth<br>☐ Ongoing pregnancy<br>☐ Miscarriage - 1st trimester (< 14 weeks)<br>☐ Miscarriage - 2nd trimester (< 22 weeks)<br>☐ Stillbirth (≥ 22 weeks and weight ≥ 500 g)<br>☐ Induced abortion<br>☐ Ectopic pregnancy<br>☐ Other
	if OTHER ➡ please specify	

2nd Pregnancy	Method	☐ Natural conception (sexual intercourse)<br>☐ ART (Assisted Reproductive Technology)<br>☐ Unknown
	Status	☐ Live birth<br>☐ Ongoing pregnancy<br>☐ Miscarriage - 1st trimester (< 14 weeks)<br>☐ Miscarriage - 2nd trimester (< 22 weeks)<br>☐ Stillbirth (≥ 22 weeks and weight ≥ 500 g)<br>☐ Induced abortion<br>☐ Ectopic pregnancy<br>☐ Other
	if OTHER ➡ please specify	

Record ID	

Pregnancy outcomes <u>NOT USING</u> their previous fertility preservation measures

3rd Pregnancy	Method	☐ Natural conception (sexual intercourse)<br>☐ ART (Assisted Reproductive Technology)<br>☐ Unknown
	Status	☐ Live birth<br>☐ Ongoing pregnancy<br>☐ Miscarriage - 1st trimester (< 14 weeks)<br>☐ Miscarriage - 2nd trimester (< 22 weeks)<br>☐ Stillbirth (≥ 22 weeks and weight ≥ 500 g)<br>☐ Induced abortion<br>☐ Ectopic pregnancy<br>☐ Other
	**if OTHER ➡ please specify**	

4th Pregnancy	Method	☐ Natural conception (sexual intercourse)<br>☐ ART (Assisted Reproductive Technology)<br>☐ Unknown
	Status	☐ Live birth<br>☐ Ongoing pregnancy<br>☐ Miscarriage - 1st trimester (< 14 weeks)<br>☐ Miscarriage - 2nd trimester (< 22 weeks)<br>☐ Stillbirth (≥ 22 weeks and weight ≥ 500 g)<br>☐ Induced abortion<br>☐ Ectopic pregnancy<br>☐ Other
	**if OTHER ➡ please specify**	

Record ID	

WHOQOL-BREF

The World Health Organization Quality of Life Brief 26-item Version

English version

Granted permission for WHO copyrighted material; request number: ***393701***

WHO REFERENCE NUMBER: ***WHO/HIS/HSI Rev.2012.03***

Instructions

This assessment asks how you feel about **your quality of life, health, or other areas of your life.** Please read each question, assess your feelings, and circle the number on the scale for each question **that gives the best answer for you.** Please keep in mind your standards, hopes, pleasures and concerns. We ask that you think about your life **in the last 2 weeks.**

		Very poor	Poor	Neither poor nor good	Good	Very good
1 (G1)	*How would you rate your quality of life?*	1	2	3	4	5

		Very dissatisfied	Dissatisfied	Neither satisfied nor dissatisfied	Satisfied	Very satisfied
2 (G4)	*How satisfied are you with your health?*	1	2	3	4	5

The following questions ask about **how much** you have experienced certain things in the last two weeks.

		Not at all	A little	A moderate amount	Very much	An extreme amount
3 (F1.4)	*To what extent do you feel that (physical) pain prevents you from doing what you need to do?*	1	2	3	4	5
4 (F11.3)	*How much do you need any medical treatment to function in your daily life?*	1	2	3	4	5
5 (F4.1)	*How much do you enjoy life?*	1	2	3	4	5
6 (F24.2)	*To what extent do you feel your life to be meaningful?*	1	2	3	4	5

		Not at all	A little	A moderate amount	Very much	Extremely
7 (F5.3)	*How well are you able to concentrate?*	1	2	3	4	5
8 (F16.1)	*How safe do you feel in your daily life?*	1	2	3	4	5
9 (F22.1)	*How healthy is your physical environment?*	1	2	3	4	5

Posttreatment 12-15 months

♀

Record ID | |

The following questions ask about **how completely** you experience or were able to do certain things in the last two weeks.

		Not at all	A little	Moderately	Mostly	Completely
10 (F2.1)	*Do you have enough energy for everyday life?*	1	2	3	4	5
11 (F7.1)	*Are you able to accept your bodily appearance?*	1	2	3	4	5
12 (F18.1)	*Have you enough money to meet your needs?*	1	2	3	4	5
13 (F20.1)	*How available to you is the information that you need in your day-to-day life?*	1	2	3	4	5
14 (F21.1)	*To what extent do you have the opportunity for leisure activities?*	1	2	3	4	5

		Very poor	Poor	Neither poor nor good	Good	Very good
15 (F9.1)	*How well are you able to get around?*	1	2	3	4	5

The following questions ask you to say **how good or satisfied** you have felt about various aspects of your life over the last two weeks.

		Very dissatisfied	Dissatisfied	Neither satisfied nor dissatisfied	Satisfied	Very satisfied
16 (F3.3)	*How satisfied are you with your sleep?*	1	2	3	4	5
17 (F10.3)	*How satisfied are you with your ability to perform your daily living activities?*	1	2	3	4	5
18 (F12.4)	*How satisfied are you with your capacity for work?*	1	2	3	4	5
19 (F6.3)	*How satisfied are you with yourself?*	1	2	3	4	5
20 (F13.3)	*How satisfied are you with your personal relationships?*	1	2	3	4	5
21 (F15.3)	*How satisfied are you with your sex life?*	1	2	3	4	5
22 (F14.4)	*How satisfied are you with the support you get from your friends?*	1	2	3	4	5
23 (F17.3)	*How satisfied are you with the conditions of your living place?*	1	2	3	4	5
24 (F19.3)	*How satisfied are you with your access to health services?*	1	2	3	4	5
25 (F23.3)	*How satisfied are you with your transport?*	1	2	3	4	5

The following question refers to **how often** you have felt or experienced certain things in the last two weeks.

		Never	Seldom	Quite often	Very often	Always
26 (F8.1)	*How often do you have negative feelings such as blue mood, despair, anxiety, depression?*	1	2	3	4	5

Thank you for your help!

FertiTOX
Fertility after gonadotoxic treatments

Posttreatment 12-15 months

Record ID	

End Study Block I

Did the patient complete the first study block (12-15 months) according to the protocol?		☐ Yes<br>☐ No
if YES ➡	Completion date	
if NO ➡	Reason for non-completion	☐ Patient withdrew consent<br>☐ Patient was lost to follow-up<br>☐ Patient died<br>☐ Other reason
	End of study date	

Min: 01-11-2023
Max: 31-12-2039
