# Supplementary material for: Evaluation of the Gonadotoxicity of Cancer Therapies to Improve Counseling of Patients About Fertility and Fertility Preservation Measures: Protocol for a Retrospective Systematic Data Analysis and a Prospective Cohort Study
Source: JMIR Res Protoc. 2024 Mar 20;13:e51145. doi: 10.2196/51145 (PMC10993117; doi:10.2196/51145)
Supplement: Multimedia Appendix 5 [file resprot_v13i1e51145_app5.pdf]

FertiTOX Fertility after gonadotoxic treatments

Posttreatment 12-15 months ♂

Record ID	

Visit

Date of posttreatment visit at 12-15 months after the end of gonadotoxic treament		*Min: 01-11-2023 Max: 31-12-2039*

Gonadotoxic treatment

Disease	*Final diagnosis, for which the fertility counselling is performed.*	*See **annexed list** with the disease list.*
if ➡ NOT LISTED		
Start of gonadotoxic treatment		*Min: 01-01-2020 Max: 31-12-2039*
End of gonadotoxic treatment		*Min: 01-01-2020 Max: 31-12-2039*
Duration of gonadotoxic treatment	*Months*	***Automatic calculation** of rounded number of months in REDCap.*
Treatment protocol *(To be added by the organizers of the study.)*		*Numbered list from 1 to 99.*
Have testicles been removed as part of cancer therapy?	▢ Yes ▢ No	
if YES ➡ How many testicles?	▢ ½ testicle ▢ 1 testicle ▢ 2 testicles	

Record ID

A
Agranulocytosis (D70)
Anal carcinoma (C21)
Anaemia - Aplastic (D60 – D61)
Anaemia - Fanconi (D61.0)
Angiosarcoma (C22.3)

B
Blasenmole ➜ **»Trophoblastic disease«**
Brain cancer:
- *Astrocytoma (C71.9)*
- *Ependymoma (C71.9)*
- *Glioblastoma (C71.9)*
- *Oligodendroglioma (C71.9)*
- *Medulloblastoma (C71.6)*
- *other (C71)*

Breast cancer:
- *benign BRCA positive (Z15.01)*
- *hormone receptor positive (Z17.0)*
- *hormone receptor negative (Z17.1)*
- *hormone receptor status not known (Z17)*
- *other (C50)*

Burkitt lymphoma (C83.7)

C
Cervix uteri carcinoma (C53)
Chondrosarcoma (C41.9)
Colitis ulcerosa ➜ **»Ulcerative colitis«**
Colon carcinoma (C18)
Crohn disease (K50)

D
Dermatomyositis (M33)

E
Endometrial carcinoma (C54)
Endometriosis (N80)
Ewing sarcoma (C40-C41)

F
Fibrosarcoma (C49.9)
Fragile X syndrome (Q99.2)

G
Galactosemia (E74.2)
Germ cell tumour - extragonadal (ICD-O-3 M906-909)

H
Hodgkin lymphoma (C81)

I
Immune thrombocytopenia (D69)

K
Keimzelltumor ➜ **»Germ cell tumour«**
Kidney cancer (C64)

L
Leiomyosarcoma NOS (ICD-O-3 M8890/3)
Leukaemia:
- *Leukaemia lymphoid - acute lymphoblastic (C91.0)*
- *Leukaemia lymphoid - chronic lymphocytic (C91)*
- *Leukaemia myeloid - acute (C92)*
- *Leukaemia myeloid - chronic (C92)*
- *Leukaemia - other forms (C91 - C95)*

Liposarcoma (C49.9)
Liver cancer (C22)
Lung cancer (C34)
Lupus erythematosus (L93)

M
Malignant fibrous histiocytoma ➜ **»Undifferentiated pleomorphic sarcoma«**
Malignant nerve sheath tumour (C47.9)
Melanoma (C43)
Mesothelioma (C45)
Mixed connective tissue disease (M35.1)
Morbus Crohn ➜ **»Crohn disease«**
Myelodysplastic syndrome (D46)
Multiple sclerosis (G35)
Myositis (M60)

N
Nasopharyngeal carcinoma (C11)
Nebennierenkrebs ➜ **»Adrenocortical carcinoma«**
Nephritis / Glomerulonephritis (N05)
Nephroblastoma ➜ **»Wilms' tumour«**
Non-Hodgkin lymphoma (C82 – C88)

O
Osteosarcoma (C41.9)
Ovary (C56):
- *borderline malignancy*
- *teratoma benign*
- *teratoma malignant*
- *other*

P
Pancreatic cancer (C25)
Placenta cancer (C58.9; D39.2) ➜ **»Trophoblastic disease«**
Pleomorphic undifferentiated sarcoma ➜ **»Undifferentiated pleomorphic sarcoma«**
Polyarteritis nodosa (M30)
Polychondritis (M94.8)
Polymyositis (M33)
Premature ovarian insufficiency (E28.3)
Prostate cancer (C61)

R
Rectal cancer (C20)
Rectosigmoid junction cancer (C19)
Renal cell carcinoma ➜ **»Kidney cancer«**
Rhabdomyosarcoma (C49.9)
Rheumatoid arthritis (M05 – M06, M08)

S
Schilddrüsenkrebs ➜ **»Thyroid cancer«**
Sharp syndrome ➜ **»Mixed connective tissue disease«**
Sickle cell disease (D57)
Sigmakarzinom ➜ **»Colon carcinoma«**
Sjögren (Sicca) syndrome (M35)
Stomach cancer (C16)
Synovial sarcoma (C49.9)
Systemic sclerosis (including: Scleroderma) (M34)

T
Takayasu arteritis (Aortic arch syndrome) (M31.4)
Testicular cancer (C62):
- *seminoma*
- *non seminomatous germ cell tumour - teratoma*
- *non seminomatous germ cell tumour - embryonal carcinoma*
- *non seminomatous germ cell tumour - yolk sac carcinoma*
- *stromal tumour - Leydig cell tumour*
- *stromal tumour - Sertoli cell tumour*
- *other histological types*

Thalassemia (D56)
Thyroid cancer (C73)
Transgender (F64)
Trophoblastic disease (O01.9)
Turner syndrome (Q96)

U
Ulcerative colitis (K51)
Undifferentiated pleomorphic sarcoma (C49)

V
Vasculitis limited to skin (L95)
Vulva carcinoma (C51)

W
Wegener granulomatosis (M31.3)
Wilms' tumour (Kidney cancer in children) (C64)

? **DISEASE NOT LISTED**

Record ID ____________

Chemotherapy

☐ Yes

if YES ➡ Protocol name (such as BEACOPPesc, CHOP, FEC, AC, etc.), medications given, dosage per cycle, number of cycles

☐ No

Radiotherapy

☐ Yes

if YES ➡ Protocol name, radiation field and applied dosage in case of pelvic radiation

☐ No

Record ID	

Immumotherapy

☐ Yes

if YES ➡ Protocol name, type of antibodies or inhibitors, dosage plus any other relevant details

☐ No

Operation / Surgery

☐ Yes

if YES ➡ Protocol name plus any other relevant details

☐ No

Other type of gonadotoxic treatment

☐ Yes

if YES ➡ Protocol name plus any other relevant details

☐ No

Record ID	

Disease status

Disease status

- ☐ Absence of tumor/disease (complete remission)
- ☐ Some tumor mass/disease left (>50% reduction = partial remission)
- ☐ Substantial tumor mass/disease left (≤50% reduction = no remission)
- ☐ Relapse (reappearance of tumor/disease)

Any important cancer treatment-related events, such as: further therapies, a second cancer, other diseases, etc.

- ☐ Yes
- ☐ No

if YES ➡ Please specify

Blood parameters

Field	Entry	Notes
**Date of blood test**		*Min: 01-11-2023*<br>*Max: 31-12-2039*
**Serum total testosterone concentration**		*Number (one decimal place), suggested range: 0.0 - 2000.0*
**Unit of the erum total testosterone value**	☐ nmol/L<br>☐ ng/dL<br>☐ microgram/L	
**Serum FSH concentration**	*IU/L*	*Number (one decimal place), suggested range: 0.0 - 160.0*
**Serum LH concentration**	*IU/L*	*Number (one decimal place), suggested range: 0.0 - 12.0*

FertiTOX
Fertility after gonadotoxic treatments

Posttreatment 12-15 months

♂

Record ID	

Sperm parameters

Date of sperm analysis (first ejaculate)		*Min: 01-11-2023* *Max: 31-12-2039*
Total sperm count in ejaculate	10^6	*Number (one decimal place), suggested range: 0.0 - 928.0*
Sperm concentration	10^6/mL	*Number (one decimal place), suggested range: 0.0 - 300.0*
Total sperm progressive motility (WHO A+B)	%	*Integer, suggested range: 5 - 85*
Proportion of sperm with normal morphology	% *If analysed in first ejaculate*	*Integer, suggested range: 0 - 30*

Legend:
A = rapid progressive
B = slow progressive
C = non-progressive
D = immotility

Fertility preservation measures

Type of fertility preservation measure performed

- ☐ None
- ☐ Sperm freezing
- ☐ Testicular tissue freezing

Several items can be chosen.

Patient satisfaction

Is the patient satisfied with the decision to have undergone fertility preservation measures or not?

- ☐ Yes
- ☐ No

How satisfied is the patient with the decision to undergo or not to undergo fertility preservation measures?

0	1	2	3	4	5	6	7	8	9	10

How satisfied is the patient with the fertility preservation counselling before the gonadotoxic treatment?

0	1	2	3	4	5	6	7	8	9	10

Slider labels:
0 *= not applicable (N/A)*
1 *= not satisfied at all*
10 *= very satisfied*

Record ID	

Pregnancy outcomes USING fertility preservation measures

Was frozen material used to achieve pregnancy?	▢ Yes	if YES ➡	Total number of pregnancies achieved after the gonadotoxic treatment using their own previous frozen material	▢ 0<br>▢ 1<br>▢ 2<br>▢ 3<br>▢ > 3
	▢ No			

1st Pregnancy		
	Frozen material	▢ Sperm freezing<br>▢ Testicular tissue freezing<br>▢ Unknown
	Status	▢ Live birth<br>▢ Ongoing pregnancy<br>▢ Miscarriage - 1st trimester (< 14 weeks)<br>▢ Miscarriage - 2nd trimester (< 22 weeks)<br>▢ Stillbirth (≥ 22 weeks and weight ≥ 500 g)<br>▢ Induced abortion<br>▢ Ectopic pregnancy<br>▢ Other
	if OTHER ➡ please specify	

2nd Pregnancy		
	Frozen material	▢ Sperm freezing<br>▢ Testicular tissue freezing<br>▢ Unknown
	Status	▢ Live birth<br>▢ Ongoing pregnancy<br>▢ Miscarriage - 1st trimester (< 14 weeks)<br>▢ Miscarriage - 2nd trimester (< 22 weeks)<br>▢ Stillbirth (≥ 22 weeks and weight ≥ 500 g)<br>▢ Induced abortion<br>▢ Ectopic pregnancy<br>▢ Other
	if OTHER ➡ please specify	

Record ID	

Pregnancy outcomes USING fertility preservation measures

3rd Pregnancy	Frozen material	☐ Sperm freezing<br>☐ Testicular tissue freezing<br>☐ Unknown
	Status	☐ Live birth<br>☐ Ongoing pregnancy<br>☐ Miscarriage - 1st trimester (< 14 weeks)<br>☐ Miscarriage - 2nd trimester (< 22 weeks)<br>☐ Stillbirth (≥ 22 weeks and weight ≥ 500 g)<br>☐ Induced abortion<br>☐ Ectopic pregnancy<br>☐ Other
	if OTHER ➡ please specify	

4th Pregnancy	Frozen material	☐ Sperm freezing<br>☐ Testicular tissue freezing<br>☐ Unknown
	Status	☐ Live birth<br>☐ Ongoing pregnancy<br>☐ Miscarriage - 1st trimester (< 14 weeks)<br>☐ Miscarriage - 2nd trimester (< 22 weeks)<br>☐ Stillbirth (≥ 22 weeks and weight ≥ 500 g)<br>☐ Induced abortion<br>☐ Ectopic pregnancy<br>☐ Other
	if OTHER ➡ please specify	

Record ID	

Pregnancy outcomes NOT USING their previous fertility preservation measures

Total number of pregnancies achieved after the gonadotoxic treatment NOT using their own previous frozen material	☐ 0<br>☐ 1<br>☐ 2<br>☐ 3<br>☐ > 3

**0 Pregnancies**	Has the patient tried to get pregnant after the end of gonadotoxic treatment?	☐ Yes<br>☐ No

**1st Pregnancy**	**Method**	☐ Natural conception (sexual intercourse)<br>☐ ART (Assisted Reproductive Technology)<br>☐ Unknown
	**Status**	☐ Live birth<br>☐ Ongoing pregnancy<br>☐ Miscarriage - 1st trimester (< 14 weeks)<br>☐ Miscarriage - 2nd trimester (< 22 weeks)<br>☐ Stillbirth (≥ 22 weeks and weight ≥ 500 g)<br>☐ Induced abortion<br>☐ Ectopic pregnancy<br>☐ Other
	**if OTHER ➡ please specify**	

**2nd Pregnancy**	**Method**	☐ Natural conception (sexual intercourse)<br>☐ ART (Assisted Reproductive Technology)<br>☐ Unknown
	**Status**	☐ Live birth<br>☐ Ongoing pregnancy<br>☐ Miscarriage - 1st trimester (< 14 weeks)<br>☐ Miscarriage - 2nd trimester (< 22 weeks)<br>☐ Stillbirth (≥ 22 weeks and weight ≥ 500 g)<br>☐ Induced abortion<br>☐ Ectopic pregnancy<br>☐ Other
	**if OTHER ➡ please specify**	

Record ID	

Pregnancy outcomes NOT USING their previous fertility preservation measures

3rd Pregnancy	Method	☐ Natural conception (sexual intercourse)<br>☐ ART (Assisted Reproductive Technology)<br>☐ Unknown
	Status	☐ Live birth<br>☐ Ongoing pregnancy<br>☐ Miscarriage - 1st trimester (< 14 weeks)<br>☐ Miscarriage - 2nd trimester (< 22 weeks)<br>☐ Stillbirth (≥ 22 weeks and weight ≥ 500 g)<br>☐ Induced abortion<br>☐ Ectopic pregnancy<br>☐ Other
	if OTHER ➡ please specify	

4th Pregnancy	Method	☐ Natural conception (sexual intercourse)<br>☐ ART (Assisted Reproductive Technology)<br>☐ Unknown
	Status	☐ Live birth<br>☐ Ongoing pregnancy<br>☐ Miscarriage - 1st trimester (< 14 weeks)<br>☐ Miscarriage - 2nd trimester (< 22 weeks)<br>☐ Stillbirth (≥ 22 weeks and weight ≥ 500 g)<br>☐ Induced abortion<br>☐ Ectopic pregnancy<br>☐ Other
	if OTHER ➡ please specify	

Record ID	

WHOQOL-BREF

The World Health Organization Quality of Life Brief 26-item Version

English version

Granted permission for WHO copyrighted material; request number: ***393701***
WHO REFERENCE NUMBER: ***WHO/HIS/HSI Rev.2012.03***

Instructions

This assessment asks how you feel about **your quality of life, health, or other areas of your life.** Please read each question, assess your feelings, and circle the number on the scale for each question **that gives the best answer for you.** Please keep in mind your standards, hopes, pleasures and concerns. We ask that you think about your life **in the last 2 weeks.**

		Very poor	Poor	Neither poor nor good	Good	Very good
**1** (G1)	*How would you rate your quality of life?*	1	2	3	4	5

		Very dissatisfied	Dissatisfied	Neither satisfied nor dissatisfied	Satisfied	Very satisfied
**2** (G4)	*How satisfied are you with your health?*	1	2	3	4	5

The following questions ask about **how much** you have experienced certain things in the last two weeks.

		Not at all	A little	A moderate amount	Very much	An extreme amount
**3** (F1.4)	*To what extent do you feel that (physical) pain prevents you from doing what you need to do?*	1	2	3	4	5
**4** (F11.3)	*How much do you need any medical treatment to function in your daily life?*	1	2	3	4	5
**5** (F4.1)	*How much do you enjoy life?*	1	2	3	4	5
**6** (F24.2)	*To what extent do you feel your life to be meaningful?*	1	2	3	4	5

		Not at all	A little	A moderate amount	Very much	Extremely
**7** (F5.3)	*How well are you able to concentrate?*	1	2	3	4	5
**8** (F16.1)	*How safe do you feel in your daily life?*	1	2	3	4	5
**9** (F22.1)	*How healthy is your physical environment?*	1	2	3	4	5

FertiTOX
Fertility after gonadotoxic treatments

Posttreatment 12-15 months ♂

Record ID	

The following questions ask about **how completely** you experience or were able to do certain things in the last two weeks.

		Not at all	A little	Moderately	Mostly	Completely
10 (F2.1)	*Do you have enough energy for everyday life?*	1	2	3	4	5
11 (F7.1)	*Are you able to accept your bodily appearance?*	1	2	3	4	5
12 (F18.1)	*Have you enough money to meet your needs?*	1	2	3	4	5
13 (F20.1)	*How available to you is the information that you need in your day-to-day life?*	1	2	3	4	5
14 (F21.1)	*To what extent do you have the opportunity for leisure activities?*	1	2	3	4	5

		Very poor	Poor	Neither poor nor good	Good	Very good
15 (F9.1)	*How well are you able to get around?*	1	2	3	4	5

The following questions ask you to say **how good or satisfied** you have felt about various aspects of your life over the last two weeks.

		Very dissatisfied	Dissatisfied	Neither satisfied nor dissatisfied	Satisfied	Very satisfied
16 (F3.3)	*How satisfied are you with your sleep?*	1	2	3	4	5
17 (F10.3)	*How satisfied are you with your ability to perform your daily living activities?*	1	2	3	4	5
18 (F12.4)	*How satisfied are you with your capacity for work?*	1	2	3	4	5
19 (F6.3)	*How satisfied are you with yourself?*	1	2	3	4	5
20 (F13.3)	*How satisfied are you with your personal relationships?*	1	2	3	4	5
21 (F15.3)	*How satisfied are you with your sex life?*	1	2	3	4	5
22 (F14.4)	*How satisfied are you with the support you get from your friends?*	1	2	3	4	5
23 (F17.3)	*How satisfied are you with the conditions of your living place?*	1	2	3	4	5
24 (F19.3)	*How satisfied are you with your access to health services?*	1	2	3	4	5
25 (F23.3)	*How satisfied are you with your transport?*	1	2	3	4	5

The following question refers to **how often** you have felt or experienced certain things in the last two weeks.

		Never	Seldom	Quite often	Very often	Always
26 (F8.1)	*How often do you have negative feelings such as blue mood, despair, anxiety, depression?*	1	2	3	4	5

Thank you for your help!

Record ID	

End Study Block I

Did the patient complete the first study block (12-15 months) according to the protocol?		☐ Yes<br>☐ No
if YES ➡	Completion date	
if NO ➡	Reason for non-completion	☐ Patient withdrew consent<br>☐ Patient was lost to follow-up<br>☐ Patient died<br>☐ Other reason
	End of study date	
		Min: 01-11-2023<br>*Max: 31-12-2039*
